# Suicidality and mood: the impact of trends, seasons, day of the week, and time of day on explicit and implicit cognitions among an online community sample

**DOI:** 10.1038/s41398-023-02434-1

**Published:** 2023-05-12

**Authors:** René Freichel, Brian A. O’Shea

**Affiliations:** 1 Department of Psychology, University of Amsterdam, Amsterdam, Netherlands; 2 School of Psychology, University of Nottingham, Nottingham, UK; 3 Department of Psychology, Harvard University, Cambridge, MA USA

**Keywords:** Human behaviour, Scientific community

## Abstract

Decades of research have established seasonality effects on completed and attempted suicides, with rates increasing in spring. Little advancements have been made to explain this phenomenon, with most studies focusing almost exclusively on the number of suicide attempts and deaths. Using more than six years of data collected among a US, UK, and Canadian online community sample (*N* > 10,000), we used newly developed Prophet forecasting and autoregressive-integrated moving average time-series models to examine the temporal dynamics of explicit and implicit self-harm cognitions. We created three groups (past suicide attempters; suicide ideation and/or non-suicidal self-injury; no previous self-harm, suicidal thoughts, or behaviors). We found a general increase of negative self-harm cognitions across the six years and seasonality effects for mood and desire to die, particularly among those who previously made a suicide attempt. Negative explicit self-harm cognitions peaked in winter (December), with implicit self-harm showing a lagged peak of two months (February). Moreover, daily negative self-harm cognitions consistently peaked around 4–5 am, with implicit cognitions again showing a lagged effect (1-hour). Limitations include the volunteer sample not being representative and the cross-sectional nature of the data being unable to separate between-subject and within-subject structural trends in the time series. Our findings show that negative explicit and implicit cognitions precede the rise in suicidal behaviors in spring. We proposed a conceptual model of seasonal suicide risk that may offer fertile ground for theoretical advancements, including implications for clinical risk assessment and public policies regarding the availability of health services.

Suicide is a major cause of mortality worldwide, and it constitutes a significant public health problem [1]. In the United States (US), suicide was the 10th leading cause of death in 2016 [2] and it has steadily increased 35% from 1999 to 2018 [3]. These trends are consistent with a general worsening of mental health in the US and the United Kingdom (UK) [4–6]. As widely noted, a history of mental illness, particularly depression, and self-harm, is strongly related to suicidal ideation and suicide attempts [7]. Identifying temporal periods with increased suicide risk and understanding how time can interact with underlying risk factors of suicide, such as past suicide attempts, is highly relevant for clinicians and researchers.

## Yearly seasonality, weekly and daily patterns of suicide and suicide attempts

Research on the impact of external time points such as traumatic events, negative or adverse life events as precipitants of suicide attempts, has been integrated into a broader discussion about seasonal patterns of suicide. Most studies examining seasonality effects report a consistent increase in suicides and suicide attempts during spring and early summer [8–10]. Importantly, little theoretical and empirical advancement has been made to explain why suicides rise in spring and early summer. Our study aimed to test a potential mechanism through which negative cognitions in winter may exert lagged effects on suicidal behavior in spring.

Seasonal fluctuations of death by suicide have been widely studied, however, much less is known about patterns concerning the days of the week and the time of the day. A study of a Danish population showed more suicide attempt admissions during weekends, both among individuals with and without mood disorders [11]. Other studies showed that suicides occurred most frequently on Sundays [12] and Mondays [13].

A less extensively studied area of research concerns the effects of the time of the day on suicides. Both attempted and completed suicides are affected by diurnal and circadian rhythms: Several studies on suicide attempts in diverse populations showed that suicide attempts occurred most frequently during evenings and nights [13, 14]. The number of completed suicides over the course of the day shows a peak during the morning hours [15, 16]. However, as noted by Preti & Miotto [15], the observed variation was based on the time of death and not the time of the suicidal act, which may precede it by a significant period.

## Temporal patterns in explicit and implicit measures of self-harm and suicide

Existing research on seasonal and time effects on suicide almost exclusively focused on completed suicides (death statistics, epidemiological studies) and suicide attempts (hospitalizations, medical records, health care data). However, the existence of temporal patterns (seasonality, day of the week, time of the day) in explicit self-report measures of self-harm and suicide remains largely unknown. Recently, labs have started using Ecological Momentary Assessment (EMA) to measure daily dynamics in suicidal cognitions (e.g., Kleiman et al. [17]; Sedano-Capdevila et al. [18]). However, while these methods provide valuable insights into within-person temporal dynamics, these assessments are primarily confined to small clinical samples over a relatively short duration. In contrast, here we use data from a large community sample over many years.

Implicit measures, a class of measures that rely on reaction times and capture more automatic cognitive biases, have shown promise in suicide research [19, 20]. Implicit associations with self-harm and suicide were shown to be robust, correlated with specific types of self-harm behaviors [21], and predictive of the occurrence of suicide ideation and future suicides [22]. Previous time-series analyses on implicit social-group attitudes showed both stability and long-term change over a 10-year period [23]. Our study is the first to examine the temporal dynamics of explicit and implicit associations in the mental health domain, specifically self-harm associations.

## Current study

Our study aims to assess long-term trends and the effects of seasons, day of the week, and time of the day on mood, desire to die, desire to self-injure, and implicit measures of self-harm (negative mood and self-harm cognitions). Our analyses aim to test the generalizability of time effects found at the level of suicide attempts and completed suicides reported in previous studies. In accordance with the literature, we predicted an increase in negative mood, explicit suicidality, and implicit self-harm associations between 2012–2018 and a peak in suicide/self-harm cognitions in spring and early summer. We predicted negative self-harm cognitions peaking towards the end of the week with a peak on Sundays or Mondays and during night hours. The unique contribution of our study is the analysis of structural, temporal trends of both explicit and implicit (cognitive risk factors) measures.

## Methods

### Data source/respondents

We used data from the Project Implicit Health (PIH) database (www.projectimplicithealth.com) and included responses between April 2012 and November 2018. All respondents voluntarily visited the PIH website and chose to complete a self-harm Implicit Association Test (IAT). We included data from participants who were residing in the US, the UK, and Canada. The final sample was 10,448 (see Supplementary Materials (SM) section 1 for more details and SM2 for details on data pre-processing). Our sample was predominantly young, female, and showed significant variance with respect to the self-reported history of self-injurious thoughts and behaviors. Notably, the proportion does not appear to differ substantially throughout the years, months, days, or hours (see Figure S1–S4).

### Procedure and choice of primary measures

After providing informed consent and selecting the ‘self-harm’ task on PIH, participants were randomly assigned to complete a self-harm IAT, where different words and images (i.e., suicide/life) were presented on a screen with attributes (i.e., me/not me). Respondents were assigned to one of four IATs: Cutting (*n* = 1143); Suicide (*n* = 2674); Death (*n* = 2712); brief Death IAT (*n* = 1449). The four IATs have different target words and images (i.e., cutting/no cutting; suicide/life; death/life) that are each presented on a screen with attributes (i.e., me/not me). For instance, the death IAT with the “death” category contains target examples of “death” (e.g., Die, Dead) and “life” (e.g., Alive, Living). The categories differ between the IATs (suicide IAT: suicide category (e.g., hanging, overdose), cutting IAT (e.g., images of cut skin and uncut skin). The selected IATs [24] measure implicit cognition about self-harm in a computerized task. Participants were instructed to distinguish and correctly classify the stimuli using two keys on a keyboard. A difference score (D-Score) was computed for each participant and represents the strength of association between two concepts (i.e., death and life) with the self. See SM2 for a detailed description of the IATs.

Three single-item explicit measures (see Table S2) assessed participants’ desire to self-injure, desire to die, and their current mood on a Likert-scale. Participants’ history of self-injury was assessed using responses from the abbreviated version of the Self-Injurious Thoughts and Behaviors Interview (SITBI) [25]. We compared three groups of respondents depending on the severity of self-injurious thoughts and behaviors (1: past suicide attempt, 2: a history of suicide ideation and/or non-suicidal self-injury, 3: no previous self-harm, suicidal ideation, or behavior).

### Time-series analysis

#### Prophet model

We used Prophet models from the *prophet* R package that was designed to forecast time series data [26]. The Prophet model adopts a Bayesian curve fitting method for forecasts with three main components: trend (non-period changes across the entire time interval), period changes (e.g., daily, weekly, yearly seasonal patterns), and effects of holidays. The Prophet model offers advantages in time series that have missing data, shifts in its trend, and strong seasonal components. Importantly, Prophet models have successfully been applied in detecting trends and daily, weekly, and yearly seasonal patterns [27], and were shown to outperform ARIMA in some instances [28]. See SM3 for the cross-validation.

#### ARIMA model

ARIMA models use three parameters to describe a time series: *p* (autoregressive terms), *d* (non-seasonal differences), *q* (moving averages terms). In seasonal time series (see SM3), seasonal ARIMA models (SARIMA) incorporate both seasonal and non-seasonal components. The ARIMA models have the advantage of dealing with temporal autocorrelations due to their flexibility [29]. Importantly, ARIMA models are commonly used in applied time-series analyses to characterize seasonality effects, such as in patterns of suicides [30].

### Data analytic plan

We used two advanced analytical approaches, namely Prophet models and ARIMA models, to examine the effect of temporal patterns (see SM3) on explicit and implicit cognitions. The Prophet models were used to characterize different temporal patterns (i.e., daily, weekly, yearly, trends across years) using all individual observations in the data. We used gold-standard ARIMA models to internally replicate the yearly seasonality effects identified by Prophet. ARIMA models have been widely used to characterize seasonality effects in suicide [31], and they allowed us to formally test whether the time series can be described using seasonal or non-seasonal parameters, a formal comparison that Prophet models cannot do.

Additional *t* tests were used as a convergent, but less conservative method of testing differences between the months with the lowest and highest outcome scores. Separate group analyses using the Prophet models are available in the SM. The pre-processed dataset and analysis scripts can be accessed via the Open Science Framework (https://bit.ly/3s3rTdh).

## Results

### Trends and seasonal patterns in explicit and implicit measures

First, we forecasted the distribution of all four outcome variables and decomposed them into trends across the years, yearly seasonality, weekly, and hourly effects. The decomposed effects shown in the following figures control for all other seasonal patterns.

#### Trends

**Fig. 1:**
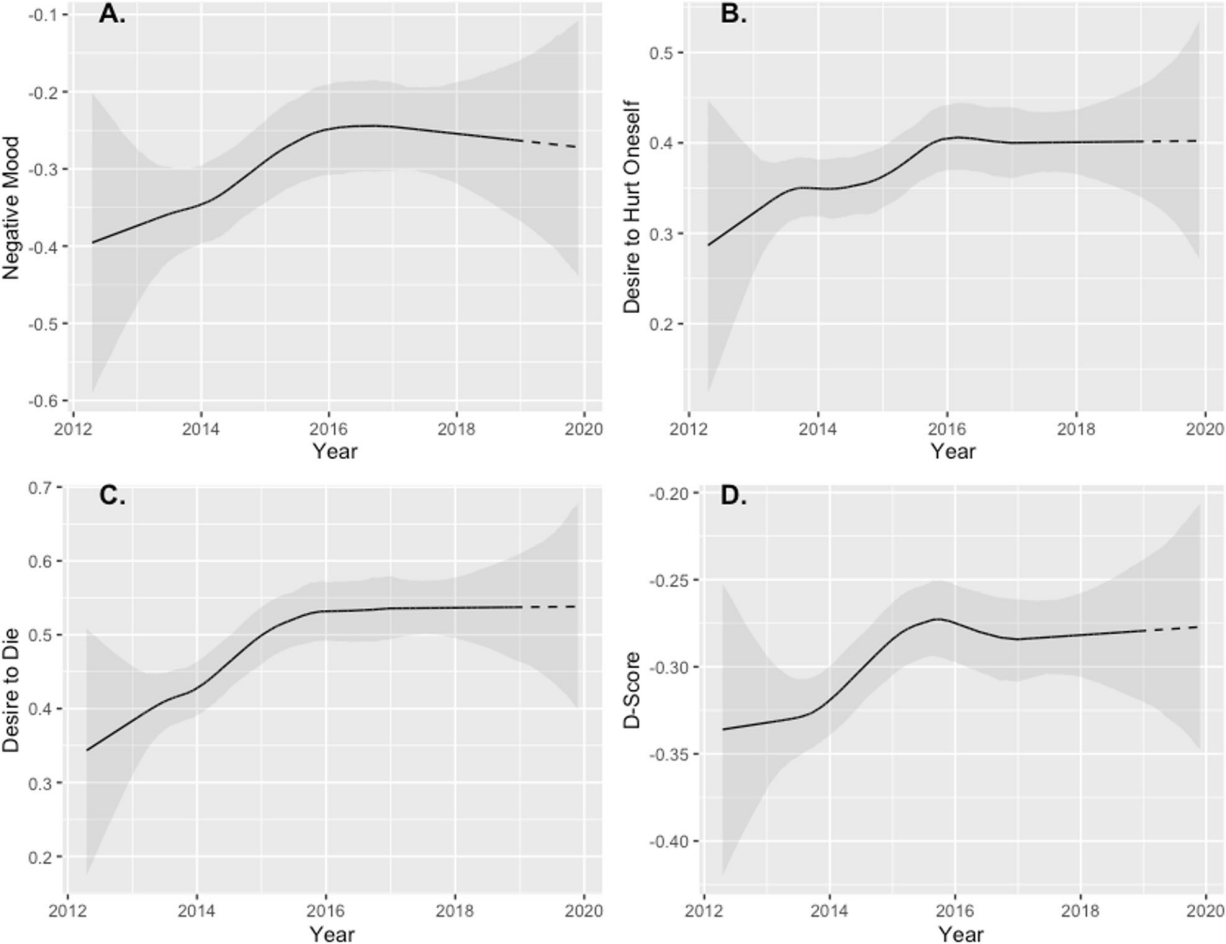
Trends decomposed from the Prophet Forecast for Mood, Desire to Hurt Oneself, Desire to Die, and the IAT D-scores. The graphs show both the trends in the training data (continuous, *N* > 7912) and the expected trend in the forecast for one year (dashed).

The Prophet models showed upwards trends in all outcome variables from 2012 to 2018 and an expected increase in the forecasted period until November 2019 for all outcome variables (see Fig. 1). The observed increases between April 2012 and November 2018 are particularly strong for both negative mood (21.66%) and the desire to die (26.56%). The increases for both the desire to self-injure (16.82%) and the IAT D-scores (13.50%) are substantially lower.

#### Yearly seasonality

**Fig. 2:**
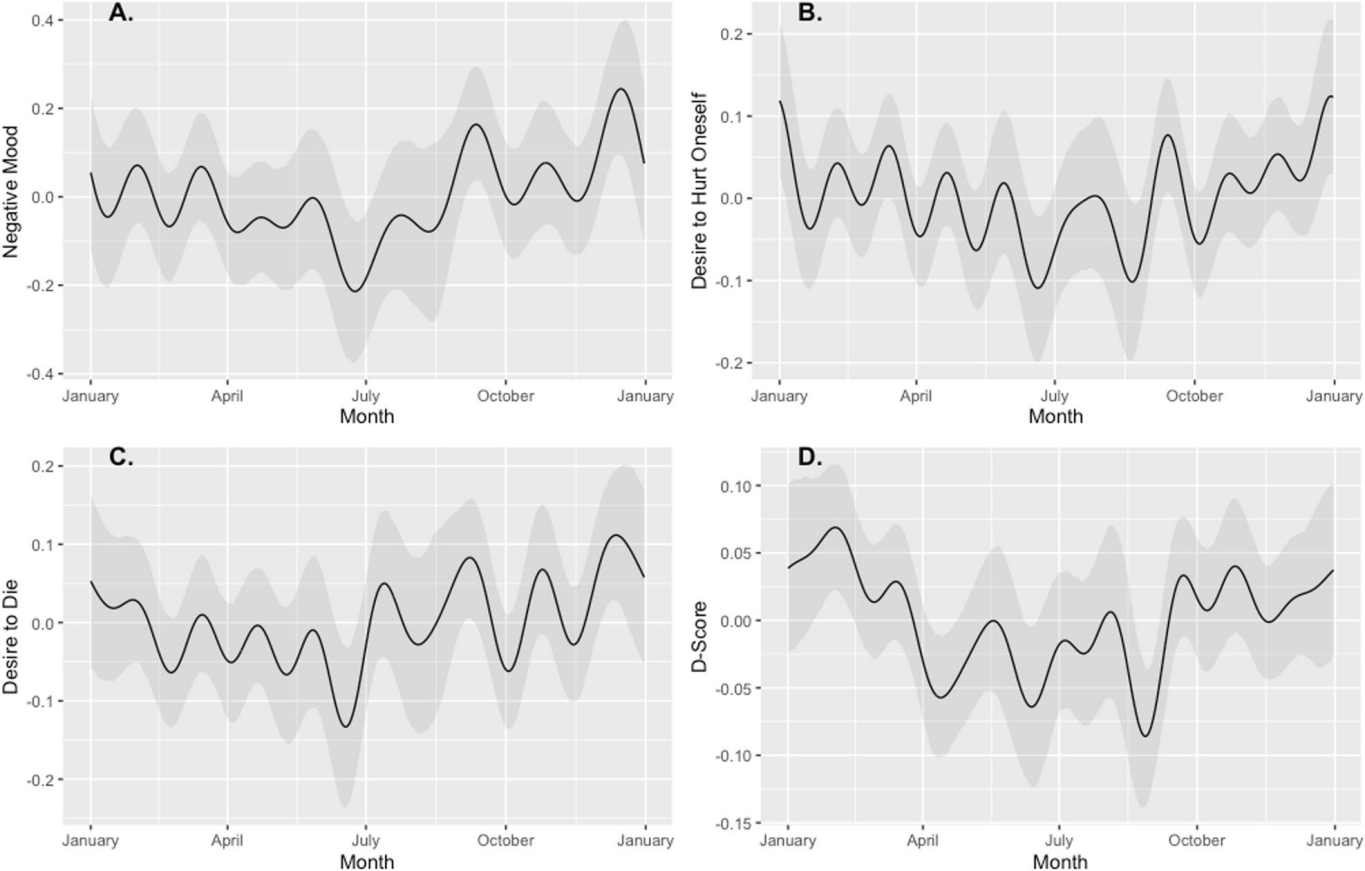
Yearly seasonality decomposed from the Prophet Forecast for Mood, Desire to Hurt Oneself, Desire to Die, and the IAT D-Scores. High scores indicate more negativity for each variable. Scores are highest in winter and lowest in summer.

The Prophet model forecasts clearly show yearly seasonal changes throughout the year (see Fig. 2). There is an apparent minimum in summer (June) and maximum in winter (December) for mood, desire to die, and desire to self-injure. For a comparative overview of the effects for the different temporal patterns, see Figures S6–S9. We observe a slightly shifted seasonal pattern in the implicit IAT D-Scores that peak in February and are lowest in September. Based on this shifted pattern, we used Granger causality tests at lags of 1, 2, and 3 months (see SM3 and Table S5). This exploratory analysis showed a significant effect of the desire to die on the IAT score, only at a lag of 2 months (*F* = 5.73, *p* < 0.01). There were no significant effects of the IAT D-Score predicting the desire to die at any of the lags, suggesting that change in the explicit desire to die likely precedes the implicit change. However, this effect was shown when all four IATs (Death, Brief Death, Suicide, Cutting) were combined, but when the cutting IAT was removed from the analysis, weaker/inconsistent effects (F = 2.60, *p* < 0.08) were observed (see Table S6).

#### Weekly effects

The effects for the day of the week (see Figure S11) were small compared to all other seasonal components (Figure S6–S9). All outcomes peaked during the beginning of the week (Monday: negative mood; desire to self-injure; Tuesday: desire to die, D-Scores) and were lowest towards the end of the week (Thursday: desire to die, desire to self-injure, Friday: negative mood, Saturday: D-Scores). Exploratory tests of Granger causality showed no significant predictions of change at lags of 1 and 2 days (see Table S5).

#### Daily effects

**Fig. 3:**
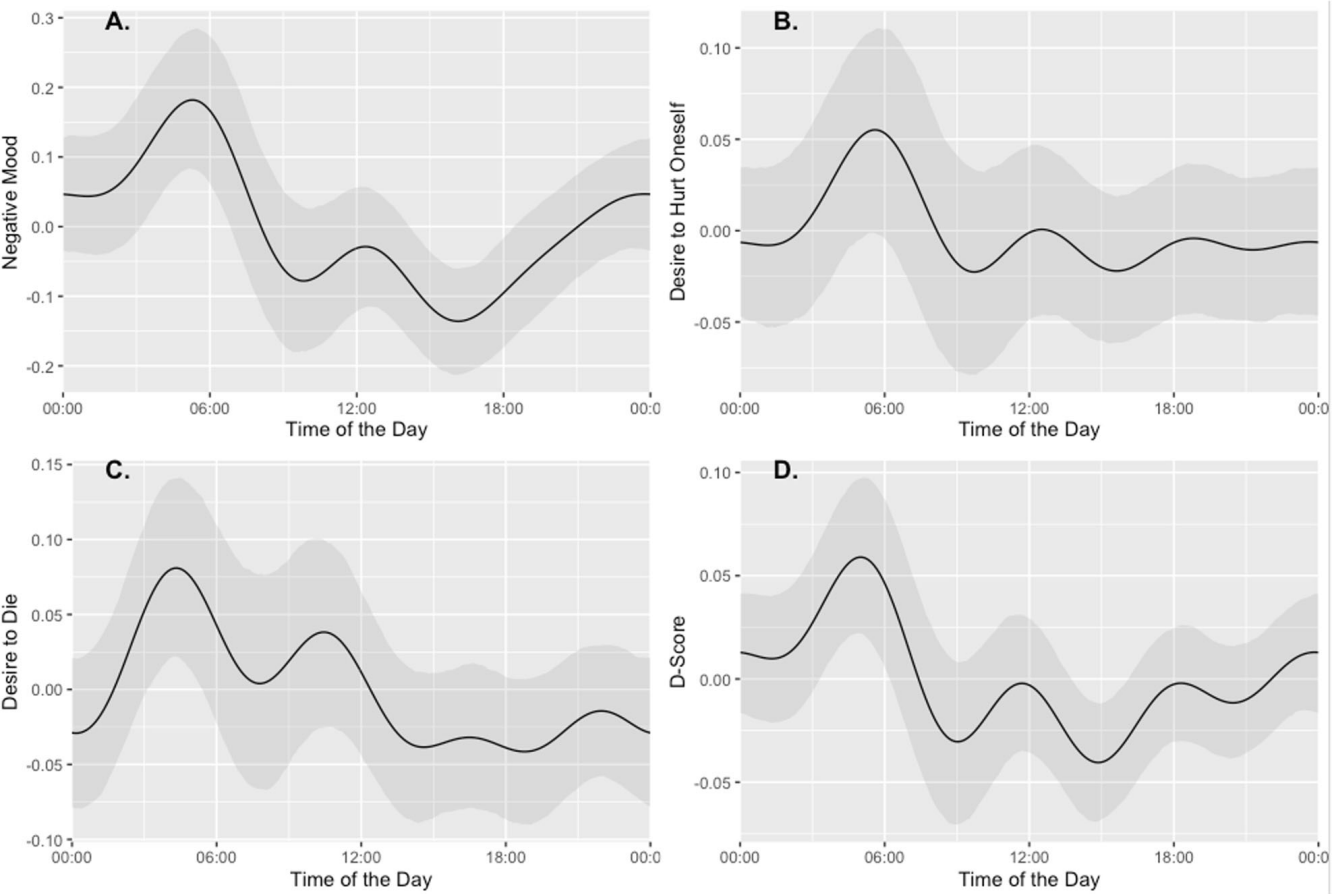
Daily seasonality decomposed from the Prophet Forecast for Mood, Desire to Hurt Oneself, Desire to Die, and the IAT D-Scores. High scores indicate more negativity for each variable. Scores are highest during night hours (peaking around 4–5 a.m.) and lowest during daylight hours.

For the daily seasonality (see Fig. 3), we observed a similar pattern in all outcomes, with peaks during the night hours (4 am–5 am) and a minimum during the afternoon/evening hours. When examining the range of the daily seasonal patterns, they are similarly impactful to both trends and yearly seasonal patterns (Figure S6–S9). The exploratory Granger tests of causality (Table S5) showed that the explicit desire to die predicts the implicit IAT D-Scores at a lag of one hour (*F* = 3.88, *p* < 0.04). The reverse direction was not significant, indicating that the change in the implicit measure likely follows changes in the explicit. However, again this result should be treated cautiously because when the cutting IAT is excluded (Table S6), weaker marginal effects were observed (*F* = 3.37, *p* < 0.07). See Table S7–S8 for the largely null or inconsistent Granger test results for how a desire to hurt and mood relates to implicit cognition.

### Group comparison in yearly seasonality effects

Our Prophet forecasts showed nested seasonality effects with a yearly seasonal pattern. Thus, we aimed to further examine how risk factors (past suicide attempt, suicide ideation, and/or non-suicidal self-injury) interact with these yearly seasonal changes. Table 1 summarizes the results of the ARIMA models that showed clear seasonality effects for mood and the desire to die in the entire sample. Importantly, these effects only remained significant in the group of respondents that reported making a suicide attempt when ARIMA models were used. See SM5 for all seasonal ARIMA models (Figure S12–S15).

**Table 1:** Results from the ARIMA models and *t* tests for all outcomes and groups.

	Entire sample *N* = 10448	Suicide attempt *n* = 3247	Self-harm/suicide ideation *n* = 3851	No self-harm *n* = 3026
Negative mood				
ARIMA	Seasonal ***	Seasonal ***	Non-seasonal	Non-seasonal
AIC	−31.83	23.29	6.63	22.82
Model	(2, 0, 0) (0, 0, 1) [12]	(0, 0, 0) (1, 0, 0) [12]	(2, 0, 0)	(3, 0, 0)
t-statistic	−4.99**	−2.20*	−2.64*	−2.90*
Mean diff. [CI]	−0.36 [−0.5, −0.22]	−0.29 [−0.55, −0.03]	−0.30 [−0.53, 0.08]	−0.38 [−0.64, −0.12]
Cohen’s *d*	−0.27	−0.22	−0.23	−0.30
Desire to hurt				
ARIMA	Non-seasonal	Non-seasonal	Non-seasonal	Non-seasonal
AIC	−147.11	−22	−70.32	−164.67
Model	(1, 0, 0)	(3, 0, 0)	(1, 0, 4)	(5, 0, 0)
t-statistic	−3.36**	−2.38*	−1.45	−0.37
Mean diff. [CI]	−0.12 [−0.21, −0.05]	−0.21 [−0.38, −0.04]	−0.08 [−0.20, 0.03]	−0.01 [−0.08, 0.05]
Cohen’s *d*	−0.18	−0.23	−0.13	−0.04
Desire to die				
ARIMA	Seasonal **	Seasonal **	Non-seasonal	Non-seasonal
AIC	−114.75	5.99	−42.08	−92.72
Model	(0,1,1) (1,0,0) [12]	(3,0,0) (0,0,1) [12]	(4,0,0)	(0,0,0)
t-statistic	−4.56**	−3.80**	−1.82	−0.18
mean diff. [CI]	−0.21 [−0.29, −0.12]	−0.37 [−0.56, −0.18]	0.12 [−0.25, 0.01]	−0.01 [−0.12, 0.10]
Cohen’s *d*	−0.25	−0.36	−0.16	−0.02
IAT D-scores				
ARIMA	Non-seasonal	Non-seasonal	Non-seasonal	Non-seasonal
AIC	−213.92	−115.75	−133.69	−149.94
Model	(0, 0, 2)	(4, 0, 0)	(4, 0, 0)	(1, 1, 1)
t-statistic	−4.59**	−1.68	−2.70*	−2.80*
Mean diff. [CI]	−0.13 [−0.18, −0.07]	−0.10 [−0.21, 0.02]	−0.12 [−0.21, −0.03]	−0.11 [−0.19, −0.03]
Cohen’s *d*	−0.26	−0.18	−0.26	−0.28

For seasonal models, we used log-likelihood ratio tests to compare the seasonal auto-ARIMA model with a non-seasonal auto-ARIMA model. The *p* values for the log-likelihood ratio tests are reported in asterisks (*≤0.05; **≤0.01, ***≤0.001). The entire sample (*N* > 10,000) included respondents with missing SITBI information. The lowest and highest month comparison differed for the different outcome variables in the *t* test analysis (negative mood, desire to die, desire to self injure: June–December; IAT D-scores: August–January).

## Discussion

Our study investigated long-term changes and seasonal patterns in negative mood, explicit and implicit measures of self-harm, and suicide among a large online community sample. Due to data collection continually running for six years, we were also able to estimate negative cognitions for the day of the week and within the day (hourly).

### Trends in negative self-harm cognitions across years (2012–2018)

The Prophet models showed clear trends in our data with an increase in respondents’ negative self-harm cognitions across the six years. This finding is consistent with previous studies showing an increase in deaths by suicide [3], anxiety and depression symptoms [5], mood disorders, and suicide-related outcomes [32]. Although the PIH database is not representative, this convergence in yearly trends between the dataset we used here and more objective metrics such as national health records indicates that this online data collection method appears to be capturing meaningful mental health trends. It is beyond the scope of the current work to explain the worsening trends; however, various explanatory factors have been put forward [6]. Interestingly, we also showed an increase in respondents’ implicit associations with self-harm across the years, suggesting that self-harm-related implicit cognition may be a convergent marker for the increase of suicide-related explicit cognitions (cf. Schimmack [33]).

### Yearly seasonality effects

The primary goal of our study was to test yearly seasonality effects in suicide-related cognitions with hypothesized peaks in spring and early summer, which would align with the seasonal patterns of completed and attempted suicide [34]. Based on visual inspection, the Prophet models appeared to show yearly seasonal patterns for all explicit and implicit measures, but no formal statistical test was available for Prophet to confirm. However, by using the ARIMA models, we could statistically confirm that seasonality effects were present for negative mood and the desire to die in the entire sample, but this effect was primarily driven by the group of respondents that reported making a previous suicide attempt.

### Weekly and daily effects

Overall, negative self-harm cognitions from this online community sample were similar to clinical observations of increases in suicidal behaviors towards the end/beginning of the week and early morning (4–5 am). The hourly estimate is consistent with less specific previous findings showing an increased number of suicide attempts and suicide deaths during night hours [14]. This finding indicates that continually crowdsourcing explicit and implicit responses may provide an inexpensive method to accurately estimate meaningful weekly and daily behavioral patterns. PIH’s platform appears to be particularly useful for studying groups that are typically difficult to recruit due to low base rates (i.e., respondents with a past suicide attempt) and at times that are traditionally difficult to acquire data (i.e., outside of regular working hours).

### Conceptual model for why negative self-harm cognitions precede suicide attempts and deaths?

**Fig. 4:**
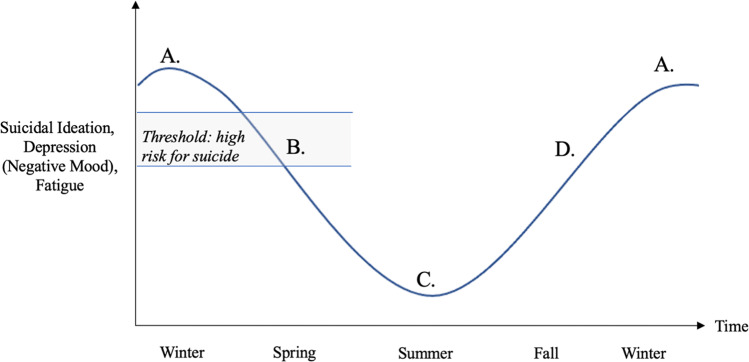
A conceptual threshold model of seasonal suicide risk. A potential timeline of events (A–D) is shown in the figure. A: Period with a high-level of suicidal ideation, fatigue, and negative mood; B: The interaction of reduced ideation, increased energy, and improved mood may put individuals within a threshold of high risk of suicide; C: Lower ideation and improved mood in summer; D: Increased ideation and lower mood after the summer period, but lower risk of suicide than B because of previously having more positive rather than negative cognitions.

We believe the most crucial finding from our analysis is the rise in negative mood and desire to die, especially among suicide attempters, occurring approximately three to four months before (December) the yearly seasonal peak of suicide attempts and deaths in spring and early summer. We propose a conceptual threshold model of seasonal suicide risk (see Fig. 4) that may explain the lag between the rise of negative self-harm cognitions in winter and the peak of suicides in spring. We hope that our proposed model can advance suicide theoretical frameworks and inspire future research with the ultimate goal of reducing seasonal suicide attempts and deaths through testing this model in longitudinal within-person designs (e.g., panel study).

First, we show that at-risk individuals for suicide gradually get more depressed, and their desire to die slowly increases as the day length becomes shorter (winter solstice). Affected individuals may become severely depressed and experience a lack of energy throughout the winter months. Essentially, this period may put them below a threshold of severe suicide risk as the high level of suicidal ideation coincides with a low level of energy. When the days get longer, brighter, and warmer, these changes likely improve an individuals’ mood. Hence, the most at-risk individuals become slightly less depressed and may gain more energy to contemplate and plan their method to attempt suicide. These improvements may result in them crossing a threshold into severe suicidal risk when a suicide attempt is most imminent. As the summer solstice approaches and passes, it is conceivable that some suicidal individuals may go above and beyond a severe suicidal risk. Therefore, a severe risk of seasonal suicides occurs when opportune conditions are met (i.e., not when an individual is the most depressed or the least depressed).

As shown in Fig. 4, our conceptual threshold model of seasonal suicide risk describes spring as a vulnerable period during which reduced suicidal ideation, increased energy, and improved mood coincide. There are two other situations in the suicide domain where similarly opportune conditions co-occur with an increased chance of suicide:

1. Some studies have shown that the risk of suicidal behavior is particularly high during the first month after starting treatment with antidepressants, especially the first nine days, compared to those who have been taking antidepressants for 90 days or more [35]. Therefore, prior to taking antidepressants, patients may be below the severe risk threshold we refer to in Fig. 4. But analogous to the weather and environmental changes improving mood, antidepressants may have a comparable impact on mood improvement, which may put them into the severe risk threshold. However, the longer patients take the antidepressants (>90 days), they may go beyond the severe risk threshold.
2. There is also evidence that suicide risk peaks shortly after being discharged from psychiatric hospitalization, and the risk reduces over time [36]. Analogous to medication and seasonal changes, it is likely that the therapy is reducing their negative cognitions, but regardless, they may still be at a severe risk until negative cognitions reduce even more.

However, for our proposed conceptual model of seasonal suicide risk, we fully acknowledge that the two above situations are not comparable with respect to the level of intensity, and the window of sensitivity, including the specific antecedents that can increase the risk of suicide. For example, for the seasonality of suicide risk, it is possible that the relative comparison between an at-risk individual and others’ level of outdoor engagement, including perceived positive interactions between individuals/groups, may become especially poignant as there are more opportunities to observe people engaging in social activities as the weather improves in spring. In contrast, the relative loss of quick access to psychiatric support may be especially apparent after one leaves a hospital. Importantly, our proposed conceptual threshold model of seasonal suicide risk merely provides a potential explanation based on the temporal variation observed in both (a) suicidal ideation and (b) negative mood. Needless to say, there may be various other complex reasons for the peak in suicide rates in spring/early summer.

### Alternative explanations for the lagged effect between negative mental cognitions and suicidal behaviors

Recent evidence suggests that within a clinical sample of participants with bipolar disorder, those participants with lifetime suicide attempts were particularly affected by climate and weather changes. The lowering of mood [37], weather changes (i.e., lower temperature and less exposure to sunshine), reduced mobility [38], and disrupted sleep and waking cycles in winter may particularly affect people with a prior history of severe mental illness, such as a past suicide attempt. Similar reasons may account for the observed seasonality in seasonal affective disorder that is characterized by periods of depression in winter and non-depressed periods in summer [39]. The strong impact of the winter season on individuals at high risk for suicide may lead to negative self-harm cognitions that persist throughout spring – perhaps unconsciously, as shown through the observed lagged IAT scores (Explicit = December peak, Implicit = February peak). These lagged effects in the implicit measure may be providing a clue as to why suicides peak in spring and early summer.

### Explicit cognitions precede implicit suicide cognitions

Changes in the explicit desire to die preceded the implicit associations (4 IATs; Death, Brief Death, Suicide & Cutting) for both hours (lags of one hour) and months (lags of two months). No lagged effects were found for the days of the week (lags of one and two days), which is consistent with our finding indicating that weekly changes only explain little variance. This lagged association between explicit and implicit suicide cognition may speak to the predictive utility of implicit cognitions as useful markers of sensitive behaviors and attitudes [19]. Thus, it is possible that explicit suicide cognitions may require some time to be internalized before they exert their lagged influence on more automatic, less reflective processes. This pattern is in line with recent studies in the social domain showing that after changes in the environment occur, implicit cognitions generally appear to parallel or follow explicit cognitions/expressions [40–42].

To the best of our knowledge, Charlesworth and Banaji (2019) are the only other authors to formally test the lagged effects between implicit (IAT) and explicit measures across social domains (e.g., racism, ageism). In contrast to our results, they found more evidence in favor of implicit biases proceeding explicit biases (6 occurrences from 28 tests) than in the other direction (3 occurrences). However, the distinction between self-harm IATs which captures self-referent information (Me versus Not Me) rather than external social categories (White People versus Black People), may be crucial. To clarify, participants may need to be consciously aware and elaborate [43] on their internal self-cognitions before the IAT can effectively capture these cognitions, while for external social cognitions, it has been speculated that the IAT is capturing the general bias in the environment (situational), not necessarily a bias ascribed to by the individual [44, 45]. These speculations warrant further research using a more diverse set of tasks examining the conditions and the specific time lag at which implicit self-referent cognitions lag, parallel, or precede explicit cognitions.

### Limitations

The findings of the present study should be interpreted in light of several limitations. First, we estimated marginal/averaged structural temporal trends using cross-sectional data from a large group of respondents. Thus, our study cannot separate between- and within-subject effects in the time series. Future research should replicate the identified patterns of explicit and implicit cognition in longitudinal designs (multiple observations from the same subject) across both short (i.e., ecological momentary assessment) and longer (i.e., panel study) time windows. Moreover, the sample in our data is disproportionately young, female, and not representative of the general population. Internet-based surveys focused on mental health may show various biases with respect to self-selection that could potentially confound the observed temporal trends. However, other Project Implicit surveys showed explicit biases that parallel trends over time to nationally representative polls [23] and are associated with meaningful regional outcomes [46, 47]. We compared three similarly sized groups that differ with respect to their clinical risk status for future suicide attempts, however, there is likely substantial heterogeneity in the group of individuals with past NSSI and suicidal ideation that we cannot capture. The responses to the items from the SITBI rely on self-report and should not be considered as established diagnoses. But regardless, given the low base rates of suicide, our sample is valuable because it acquired a high proportion of suicide attempters (38%). Lastly, the reasons for suicidal ideation and suicide attempts are the result of a complex interaction between many factors that cannot be explained solely by temporal patterns.

### Concluding comments

Our study is the first to describe temporal patterns and long-term trends in explicit and implicit measures of self-harm and suicide. Despite significant advances in the last decade in understanding the prospective utility [22] and assessment (i.e., decomposition of life- and death-associations, O’Shea et al. [48]) of implicit suicide cognitions, our study adds an investigation of temporal patterns to the literature. Our work extends both epidemiological research on suicide deaths and clinical research on psychiatric inpatients and healthcare data by using a large online sample. Using state-of-the-art time-series models, we show that (a) people with a past suicide are affected by seasonal changes, (b) there appears to be a latency between the peak of explicit and implicit suicide cognition in winter and the peak in suicide attempts and suicide deaths in spring, (c) explicit suicide cognition (peak in December) may precede implicit self-harm associations (peak in February), and (d) explicit and implicit cognitions towards self-harm and mood are strongest during late night/early morning hours. Our findings have implications for clinicians in their risk assessment of potential suicide attempts for patients at-risk and policy-makers regarding the allocation of protective services.

## Supplementary information

Supplemental Material
