# Supplementary material for: Suicidality and mood: the impact of trends, seasons, day of the week, and time of day on explicit and implicit cognitions among an online community sample
Source: Transl Psychiatry. 2023 May 12;13:157. doi: 10.1038/s41398-023-02434-1 (PMC10175253; doi:10.1038/s41398-023-02434-1)
Supplement: Supplementary file 1 — Supplemental Material [file 41398_2023_2434_MOESM1_ESM.docx]

**Suicidality and Mood: The Impact of Trends, Seasons, Day of the Week, and Time of Day on Explicit and Implicit Cognitions Among an Online Community Sample**

**Supplementary Material**

**Table of Content**

[**Supplemental Section 1: Study and Sample Characteristics** 2](#_Toc123908861)

[**Supplemental Section 2: Materials** 7](#_Toc123908862)

[**Supplemental Section 3: Analytical Strategy** 11](#_Toc123908863)

[**Supplemental Section 4: Prophet Models** 16](#_Toc123908864)

[**Supplemental Section 5: Auto-ARIMA Models** 24](#_Toc123908865)

[**Supplemental Section 6: Group Means and Comparisons** 29](#_Toc123908866)

[**Supplemental Section 7: Granger Models of Predictive Causality** 36](#_Toc123908867)

## Supplemental Section 1: Study and Sample Characteristics

**Table S1**

*Demographic and Diagnostic Characteristics of our Sample*

| Variable | Sample 1:  US ZIP code  & UK (Prophet Models Only) | Sample 2:  US no ZIP code & Canada |
| --- | --- | --- |
| Number of respondents  Age in years (M, SD) | 7978  27.01 (10.93) | 2470  25.79 (10) |
| Sex (% female) | 71.03 | 70.40 |
| Race (%)  White  Asian  Black  Native American  Multiple Selection  Other/unknown  Ethnicity (%)  Hispanic or Latino | 76.83  5.42  2.40  2.28  7.01  6.06  9.95 | 72.16  9.19  0.95  2.23  7.95  7.52  9.28 |
| Residence (%)  United States  United Kingdom  Canada | 91.84  8.16 | 74.09  0  25.91 |
| Citizenship (%) |  |  |
| United States  Non-United States  Education (%)  No high school graduation | 82.00  18.00  5.97 | 64.98  35.02  8.45 |
| High school graduation | 10.24 | 15.56 |
| College, no bachelors | 44.96 | 38.12 |
| Bachelors, graduate school | 23.11 | 23.71 |
| Graduate degree | 15.73 | 14.16 |
| History of Self-Injurious Thoughts and Behaviors (%) |  |  |
| No history of self-harm or suicide | 32.07 | 31.25 |
| Previous suicide plan or non-suicidal self-injury | 38.04 | 39.33 |
| Lifetime suicide attempt | 29.89 | 29.42 |

**Figure S1**

*Number of Respondents with a History of Suicide and Self-Harm across the Years*


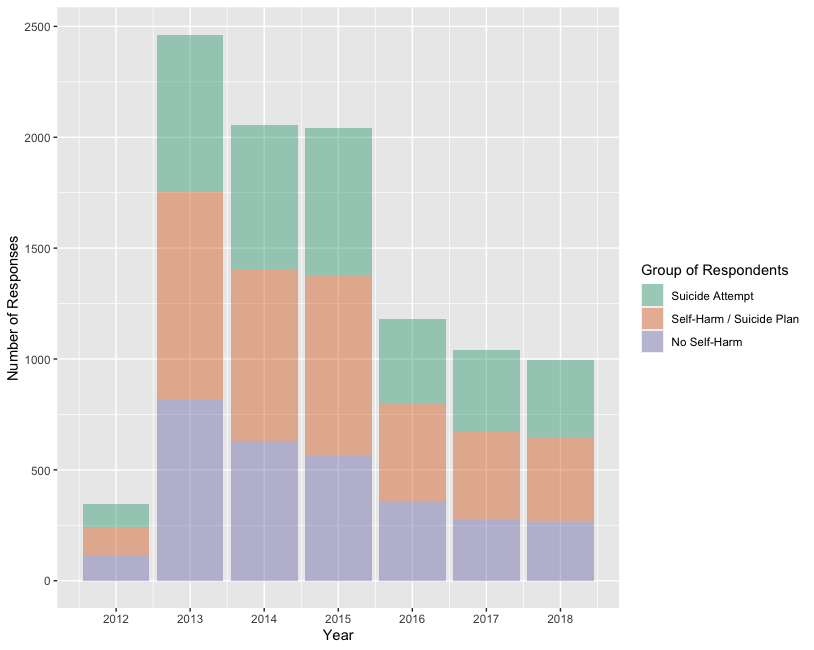


**Figure S2**

*Number of Respondents with a History of Suicide and Self-Harm across different Months of the Year*


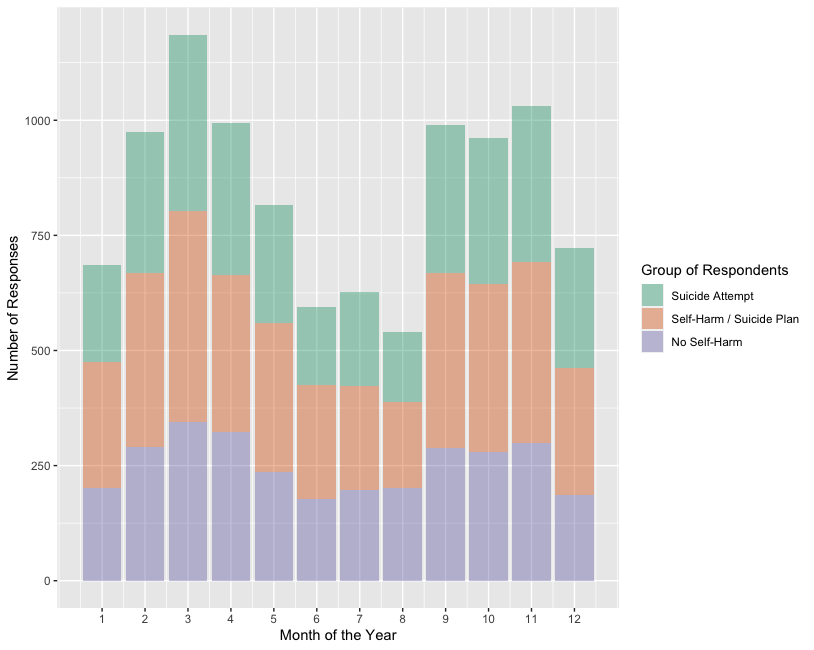


**Figure S3**

*Number of Respondents with a History of Suicide and Self-Harm at Different Days of the Week*


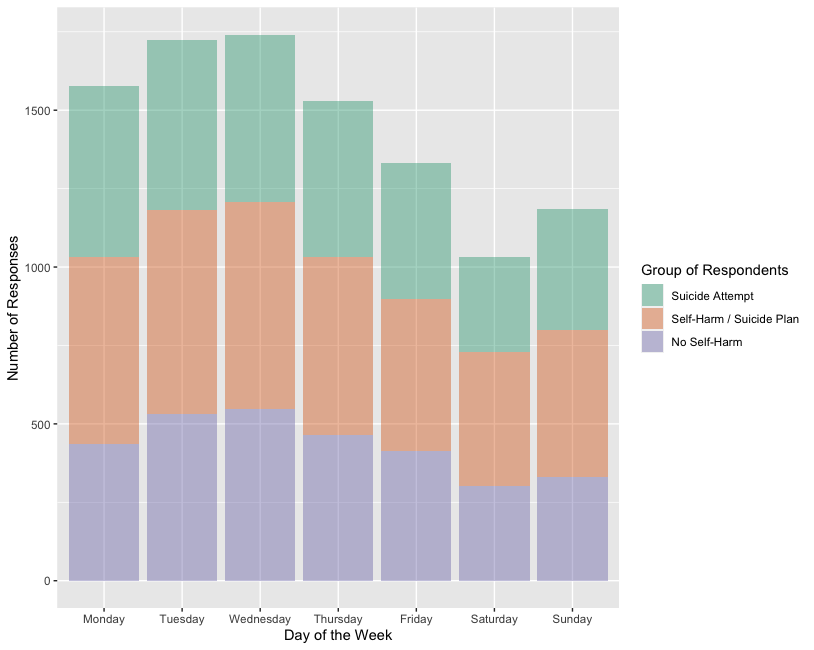


**Figure S4**

*Number of Respondents with a History of Suicide and Self-Harm at Different Times of the Day*


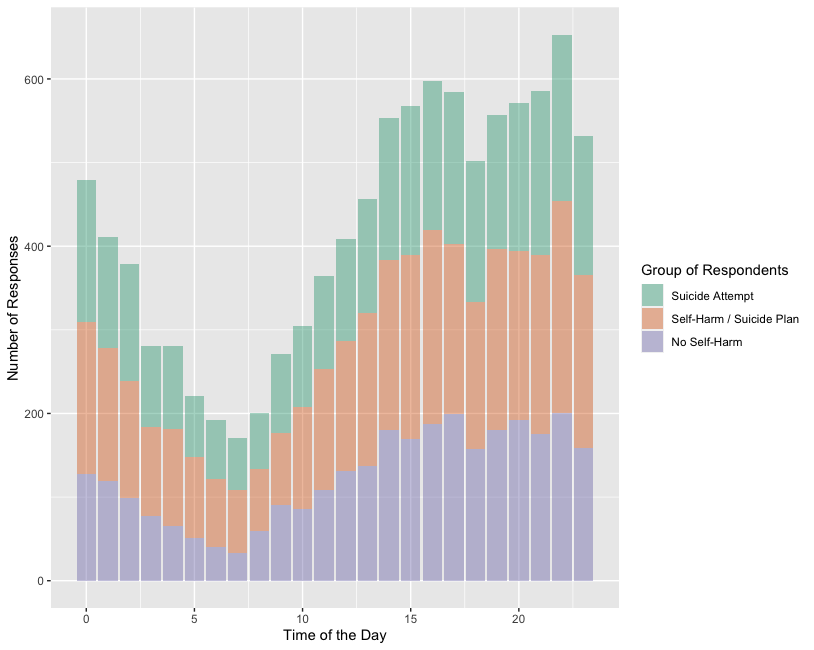


## Supplemental Section 2: Materials

**Implicit Association Tests**

Respondents were assigned to one of four IATs^1^: Cutting (*n* = 1143); Suicide (*n* = 2674); Death (*n* = 2712); brief Death IAT (*n* = 1449). The four IATs have different target words and images (i.e., cutting/no cutting; suicide/life; death/life) that are each presented on a screen with attributes (i.e., me/not me). For instance, the death IAT with the “death” category contains target examples of “death” (e.g., die, funeral) and “life” (e.g., live, survive). The categories differ between the IATs (suicide IAT: suicide category (e.g., hanging, overdoes), cutting IAT (e.g., images of cut skin and uncut skin). The IATs, explicit measures, and sociodemographic questions were presented in a random order. All explicit measures and sociodemographic questions were identical across the four IATs.

The selected IATs measure implicit cognition about self-harm in a computerized task. By pressing a left (i.e., “Life-Me”) or right (i.e., “Suicide-Not Me”) keyboard key, participants are instructed to distinguish and correctly classify different self-harm-related stimuli (i.e., cutting, suicide) from non-self-harm stimuli (i.e., life) into the appropriate categories as fast and as accurately as possible. Halfway through the task, the key assignments switch, resulting in new attribute pairings for the left (i.e., “Suicide-Me”) and right (i.e., “Life-Not Me”) keyboard keys. A red “X” appears if participants incorrectly assigned the target word. The brief death IAT is shorter than the IAT and only presents two categories on the screen without the “not me” category^2^. Respondents are instructed to use the “I” button to classify the stimuli that match the category labels and the “E” button for stimuli that do not match the labels. This shorter and simpler version of the death IAT was previously shown to be reliable and valid in distinguishing between suicide attempters and non-attempters^3^.

The IATs were scored using the most common method described by Greenwald and colleagues (2003)^4^. A standardized difference score (D-Score) was computed for each participant who fully completed the IAT. Essentially, this score represents the strength of association between two concepts (i.e., death and life) and me^2^. In all tests, positive D-Scores reflect the self-harm and me associations, while negative scores indicate the non-self-harm (i.e., life) and me associations.

To exclude responses with a high error rate or too fast responses, we used the criteria described in Glenn et al.^5^ that are slightly stricter than the recommendations from Greenwald et al.^4^: The IAT scores of participants were removed if their reaction times (RT) or error rates passed a threshold in critical blocks (> 25% of RT faster than 300ms; > 40% trial errors) or overall (> 10% of RT faster than 300 ms; > 30% trial errors).

**Explicit Self-Report Measures**

We selected three explicit measures (see Table S2) that were given to participants before and after each IAT to examine potential iatrogenic effects^6^. To increase the reliability, we average the pre- and post-IAT measures. Sociodemographic information (i.e., age, gender, country of residence, race, ethnicity) was collected from participants.

**Table S2**

*Items to assess Iatrogenic Effects and their respective Scale and Measured Construct.*

| Item | Scale | Construct |
| --- | --- | --- |
| How would you rate your mood right now? | -3 (Extremely Positive)  -2 (Moderately Positive)  -1 (Slightly Positive)  0 (Neutral)  1 (Slightly Negative)  2 (Moderately Negative)  3 (Extremely Negative) | Mood |
| How much do you want to hurt yourself right now? | 0 (Not at all)  1 (Slightly)  2 (Moderately)  3 (Strongly)  4 (Extremely) | Desire to self-injure / Desire to hurt oneself |
| How much do you want to die right now? | 0 (Not at all)  1 (Slightly)  2 (Moderately)  3 (Strongly)  4 (Extremely) | Desire to die |

**History of self-injurious thoughts and behaviors**

The Self-Injurious Thoughts and Behaviors Interview (SITBI) was shown to be a reliable tool to assess self-injurious thoughts and behaviors^7^. We used three items from the entire interview to assess a history of suicide attempts (“Have you ever made an actual suicide attempt, where you wanted to kill yourself, even just a little?”), a past suicide plan (“Have you ever actually made a plan to kill yourself?”), and a history of non-suicidal self-injury (“Have you ever done anything to purposely hurt yourself without wanting to die (for example cutting or burning your skin)?”). Participants were asked to respond to these questions on a dichotomous scale (Yes / No).

## Supplemental Section 3: Analytical Strategy

**Prophet Models**

All analyses using Prophet models were based on sample 1 that includes respondents with accurate time and date information. We always used the maximum number of valid responses for the analyses of the respective outcome variables. Thus, the sample size slightly differs for the outcomes: negative mood (*n* = 7912), desire to die (*n* = 7927), desire to hurt oneself (*n* = 7923), and the IAT D-Scores (*n* = 7978).

To obtain accuracy estimates for our Prophet forecast, we used a cross-validation procedure with independent training (April 2012 to November 2017) and test sets (December 2017 to November 2018). For the final forecast of all outcome measures, we used all available data from April 2012 to November 2018 to forecast the one-year period until November 2019 (testing set). We chose to forecast a one-year period as the validation set and as a consequence, all forecast accuracy estimates refer to a one-year period.

To assess our forecasts, we used both the root mean square error (RMSE) and the mean absolute error (MAE) as two commonly used metrics that measure the differences between observed ($y_{t}$) and predicted values ($\hat{y_{t}}$) at every time point (t) across the entire interval (T).

$$MAE=\frac{1}{T} \sum_{t = 1}^{T} \left| y_{t}- \hat{y_{t}} \right|$$

$$RMSE=\sqrt{\frac{1}{T} {\sum_{t = 1}^{T} \left| y_{t}- \hat{y_{t}} \right|}^{\begin{aligned} \\ 2 \end{aligned}}}$$

The RMSE computed to evaluate the Prophet forecast can be interpreted as the standard deviation of the unexplained variance based on the prediction. Importantly, the RMSE is in the same unit of measurement as the outcome variable (e.g., rating scale for desire to die: 0-4) with lower RMSE values indicating a better model fit. Similarly, the MAE measures the average magnitude of error in the prediction – indicating a good fit of the Prophet models to all outcome variables.

In our analysis, we aimed to validate our forecasts for a one-year period (15% of available data) that rely on models that were trained on 85% of all available data (April 2012 -November 2017). Thus, this partition allowed for a good model parameter estimation. The changepoints in the trend were automatically selected and the maximum number of changepoints was set to 25 which is a commonly used specification to capture trend changes in a time series. As there was no known saturating point in the trends, we only allowed prophet models with a linear growth. The additive prophet model was estimated with a pre-defined order of the Fourier terms (yearly: 10, weekly: 3, daily: 4). Prophet uses a posterior probability estimation (MAP) method or full Bayesian statistical inference that includes Markov Chain Monte Carlo (MCMC) sampling. At a given time, the estimated Prophet seasonal patterns are the sum of the respective trend, yearly, weekly, and daily effects.

**ARIMA Models**

Non-seasonal ARIMA models use three parameters (p (autoregressive terms), d (non-seasonal differences), q (moving averages terms)) to describe a time-series:

ARIMA model parameters: (p, d, q)

Seasonal ARIMA models contain several additional parameters, including P (seasonal autoregressive terms), D (seasonal differences), Q (seasonal moving-average terms), and S (periodic terms). In our analysis using ARIMA, the occurrence of variations in the outcomes is over the monthly period (S = 12).

SARIMA model parameters: (p, d, q) × (P, D, Q) S

Given the irregular intervals between responses for certain times of the day and missing responses on weekdays during certain months, we aggregated the data for every month for the analyses with ARIMA models. Given the large sample size in our data, the aggregated monthly estimates rely on a moderate number of observations (average per month for the entire sample: 132.66) with at least 38 observations on average per month within the different groups of respondents (see Table S4).

We used the auto-ARIMA function and explicitly allowed seasonal components to select the model parameters that defined the model order for the forecast. The auto-ARIMA function will select a seasonal model with the respective components (P, D, Q, S) if there are seasonal yearly effects in the data. We used log-likelihood ratio tests to compare seasonal ARIMA models with non-seasonal ARIMA models to test whether clear seasonality effects were present. The advantage of using the auto-ARIMA function is that all model parameters are automatically selected based on the overall model fit, as indicated by the Akaike information criterion (AIC).

Using the Augmented Dickey-Fuller test, the auto-ARIMA model automatically chooses the order of first differencing required to make the time series stationary. We used a non-stepwise auto-selection of models, thus searching over all possible model specifications. To train the ARIMA models, we used data from all months starting in December 2012 until November 2018 to ensure the models use the maximum amount of information to achieve predictive power.

**Two-sided T-Tests**

Using two-sided t-tests served as a simple statistical method to ensure that the seasonality effects manifest themselves in a statistically significant overall difference in the outcome scores between months with the yearly minimum and the yearly maximum. We controlled for the false discovery rate using the method by Benajmini and Hochberg (1995) in the t-tests that were used for multiple comparisons of two months within the three groups and four outcome measures.

First, we collapsed across the groups and years to determine the months for which the outcome variables reach their minimum and maximum. For mood, desire to die, and the desire to hurt oneself, we see a clear pattern with the highest mean scores in December and the lowest in June. The implicit D-Scores showed a slightly shifted pattern with a peak in January and a minimum in August. Thus, we compared the scores on all outcomes and groups during the peak in the summer month (June for mood, desire to die, desire to hurt oneself; August for IAT D-Scores) with the lowest point during the winter months (December for mood, desire to die, desire to hurt oneself; January for IAT D-Scores). We expected the t-tests to show significant differences in those groups and outcomes where the ARIMA models detected seasonality effects.

**Granger Models of Predictive Causality**

In an exploratory fashion, we aimed to examine the temporal relationship between changes in explicit and implicit negative cognitions. As three of the selected IATs primarily focus on death and suicide, we exclusively tested directed temporal relationships between desire to die and the IAT D-Score at the level of months, weekdays, and hours. Granger causality tests have been successfully used to examine whether changes in explicit social-group attitudes precede changes in implicit attitudes or vice versa^9^. However, little is known about the relationship for suicide cognitions. Using Wald tests, we compared restricted prediction models (e.g., desire to die is explained by lags of desire to die) with unrestricted prediction models (e.g., desire to die explained by lags of desire to die and the lags of implicit IAT D-Scores). To rule out reverse causation, we always tested both directions of change to examine whether a) explicit desire to die precedes the implicit association with death/suicide and b) implicit association precedes the explicit desire to die. When only one direction is significant, then this finding indicates the potential lagged effects with changes in one variable preceding another.

**Data Analytic Plan**

To obtain accurate time and date information, we converted participants’ local time and date to Eastern Standard Time using the zip-code and country of residence information. For our analyses we divided the sample based on the availability of accurate time and date information: We included participants who provided valid zip code information in the US (*n* = 7327) and participants from the UK (*n* = 651) in our analyses with the Prophet models (sample 1). As the Prophet model examines seasonal patterns on the level of times of the day and days of the week, accurate time and date information are required. MCMC sampling was used to estimate uncertainty intervals.

For our analyses with the ARIMA models, we included all participants (combined samples 1 and 2, *N* = 10448) from the US, UK, and Canada as we aggregated the data to monthly intervals to obtain more accurate estimates. Thus, given the aggregation to monthly intervals, it was not necessary to convert the time and date information of respondents with a residence in Canada based on their time zone information. To replicate the yearly patterns found in the Prophet models using a different methodology, we selected ARIMA models that allowed us to statistically test whether the time series can be described using seasonal or non-seasonal parameters.

## Supplemental Section 4: Prophet Models

**Table S3**

RMSE and MAE for Training and Test Set Prediction of Mood, Desire to Die and IAT D-Scores based on the Full Bayesian Estimation

|  | Mood | Desire to Hurt | Desire to Die | IAT D-Score |
| --- | --- | --- | --- | --- |
| **Test Set** |  |  |  |  |
| RMSE | 1.36 | 0.75 | 0.86 | 0.45 |
| MAE | 1.14 | 0.54 | 0.68 | 0.36 |
| **Training Set**  RMSE  MAE | 1.39  1.15 | 0.72  0.53 | 0.83  0.63 | 0.49  0.40 |

Note. Both RMSE and MAE are averages referring to the one year forecast with the trained model in the test set.

**Figure S5**

*Trends for All Outcome Variables based on the Prophet Model Forecast using the MAP Estimation Method*


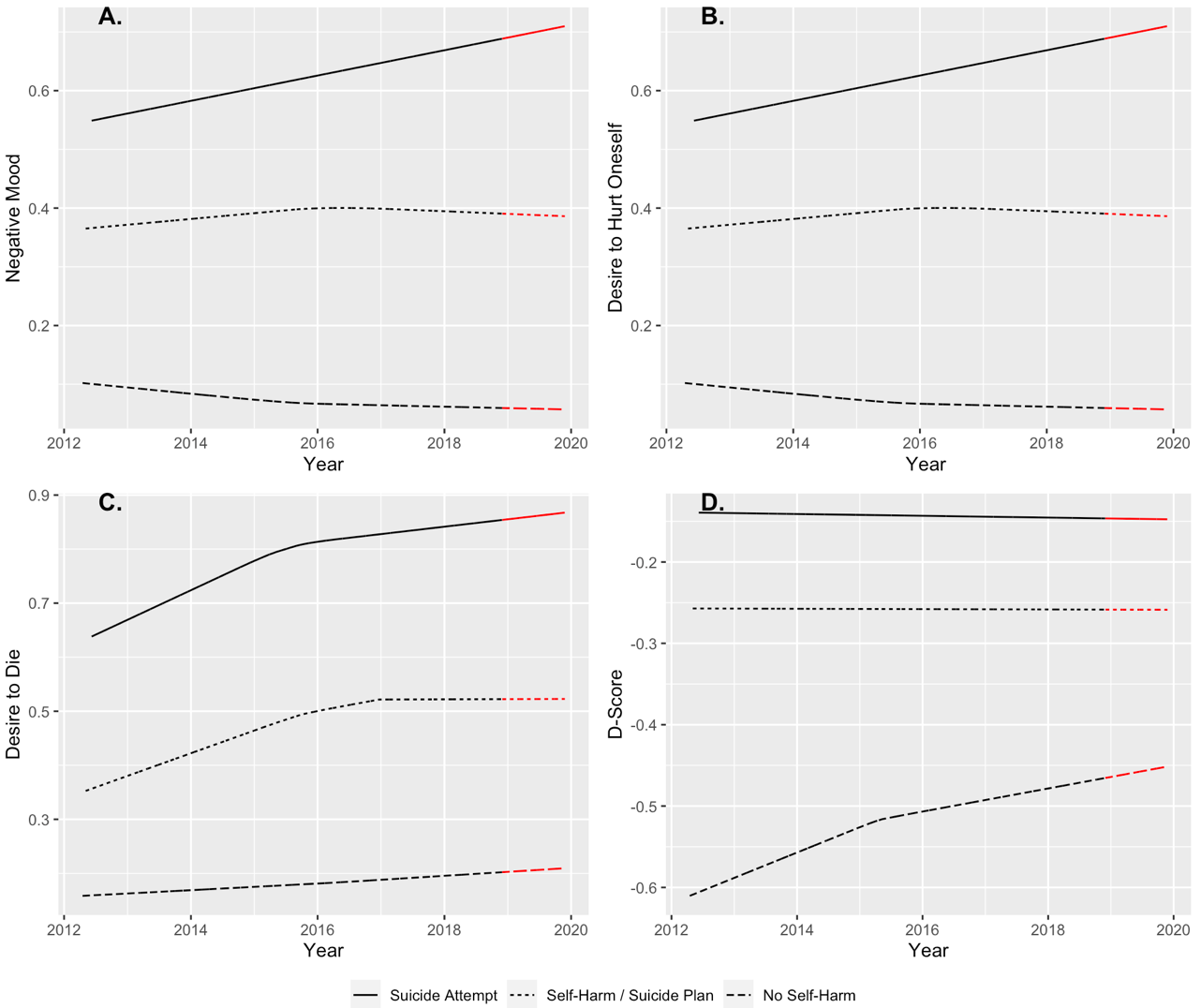


As shown in Figures S6-9, relative to the yearly seasonality effects, we observe comparably strong daily variation in negative cognitions, while weekly variation was the weakest.

**Figure S6**

*Yearly, Weekly and Daily Seasonal Patterns for Negative Mood Based on Full Bayesian Inference with Markov Chain Monte Carlo Sampling*

**
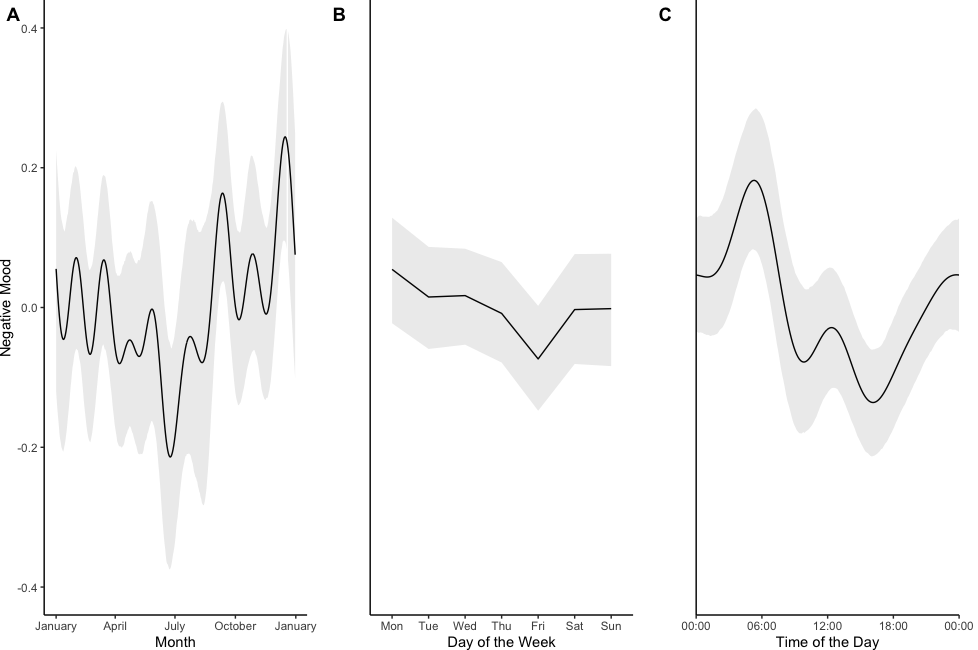
**

*Note.* The graphs show the yearly (A), weekly (B) and daily (C) seasonal patterns for the negative mood on the same scale.

**Figure S7**

*Yearly, Weekly and Daily Seasonal Patterns for Desire to Die Based on Full Bayesian Inference with Markov Chain Monte Carlo Sampling*

**
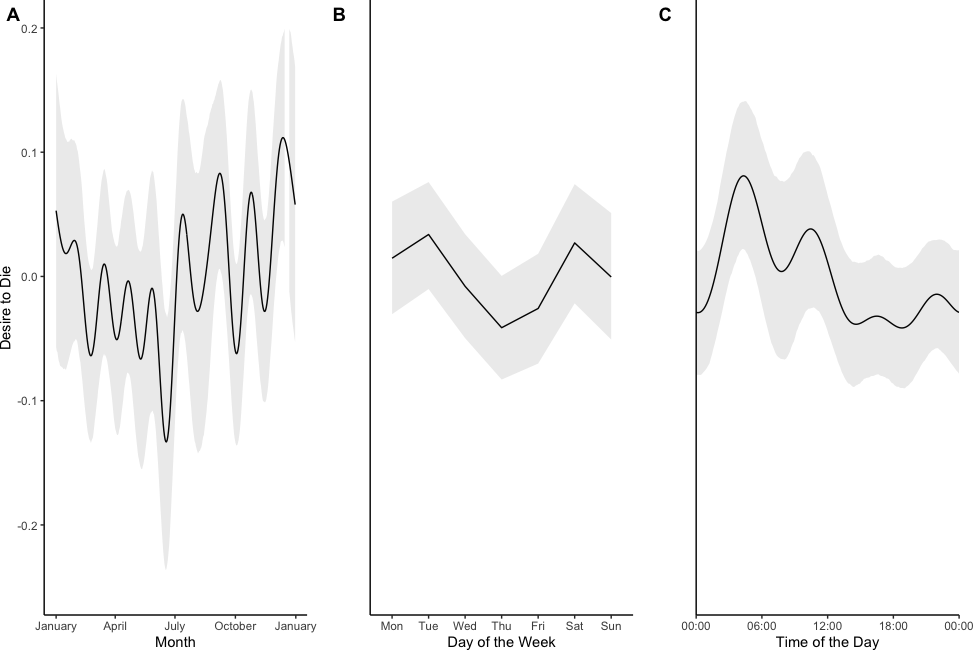
**

*Note.* The graphs show the yearly (A), weekly (B) and daily (C) seasonal patterns for the desire to die on the same scale (-0.15 to 0.15).

**Figure S8**

*Yearly, Weekly and Daily Seasonal Patterns for Desire to Hurt Oneself*


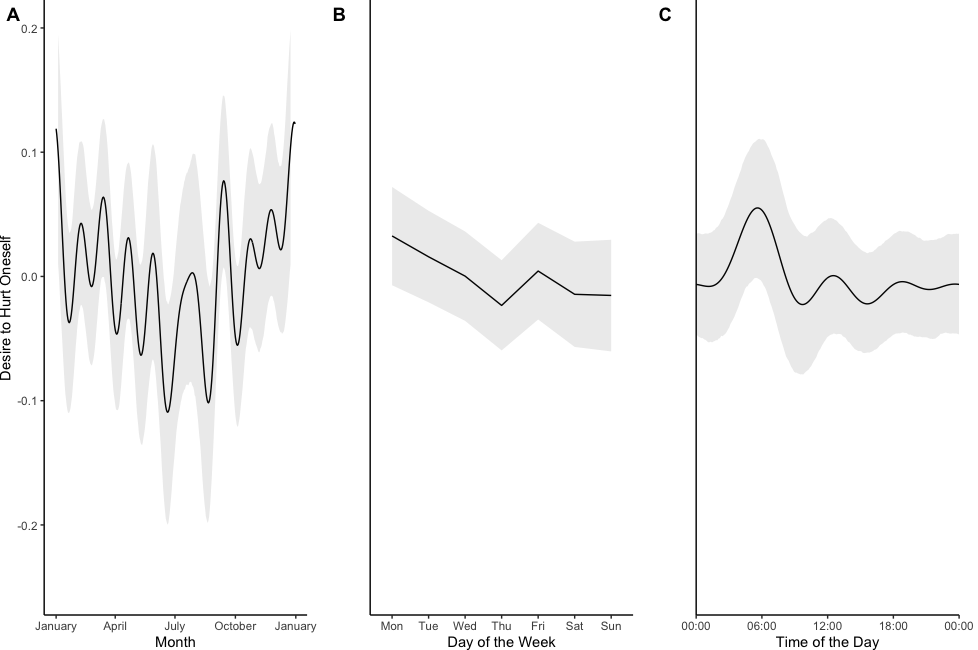


*Note.* The graphs show the yearly (A), weekly (B) and daily (C) seasonal patterns for the desire to hurt oneself on the same scale (-0.15 to 0.15).

**Figure S9**

*Yearly, Weekly and Daily Seasonal Patterns for the IAT D-Scores Based on Full Bayesian Inference with Markov Chain Monte Carlo Sampling*


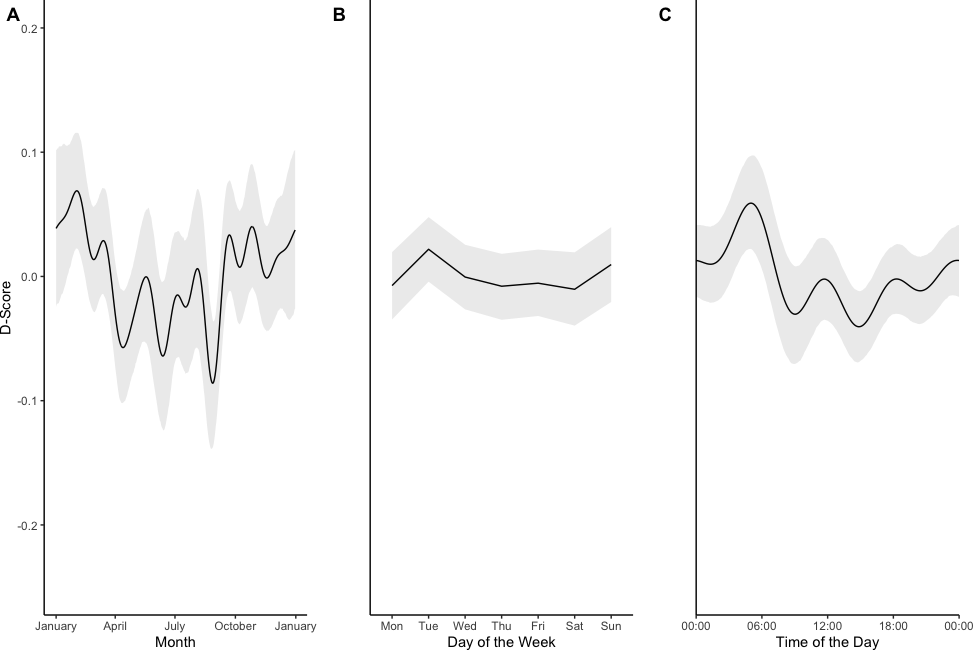


*Note.* The graphs show the yearly (A), weekly (B) and daily (C) seasonal patterns for the IAT D-Score on the same scale (-0.10 to 0.10).

**Figure S10**

*Yearly Seasonality for All Outcome Variables Based on the Prophet Model Forecast Based on MAP Estimation Method*


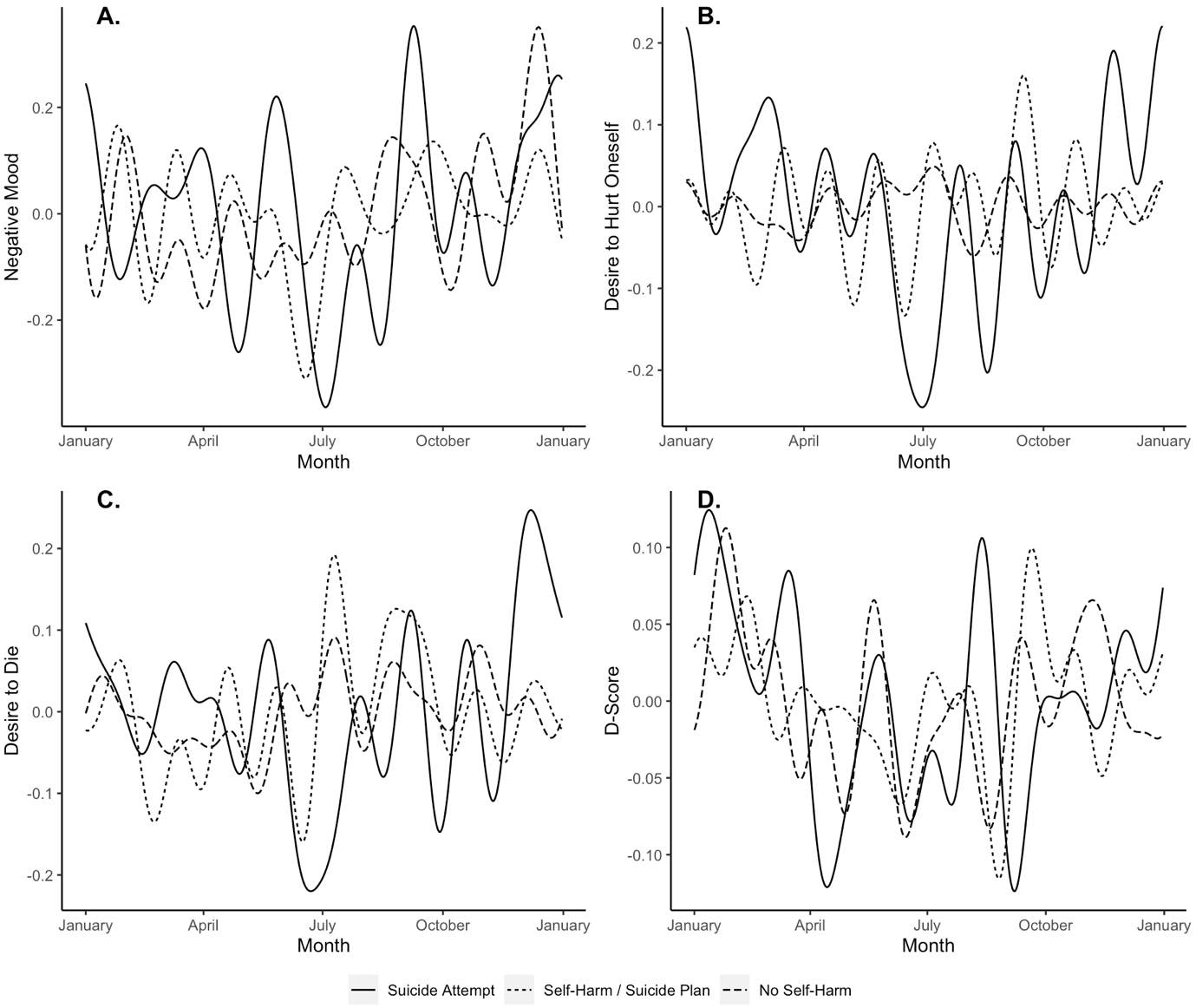


**Figure S11**

*Weekly Seasonality Decomposed from the Prophet Forecast for Mood, Desire to Hurt Oneself, Desire to Die and the IAT D-Scores*


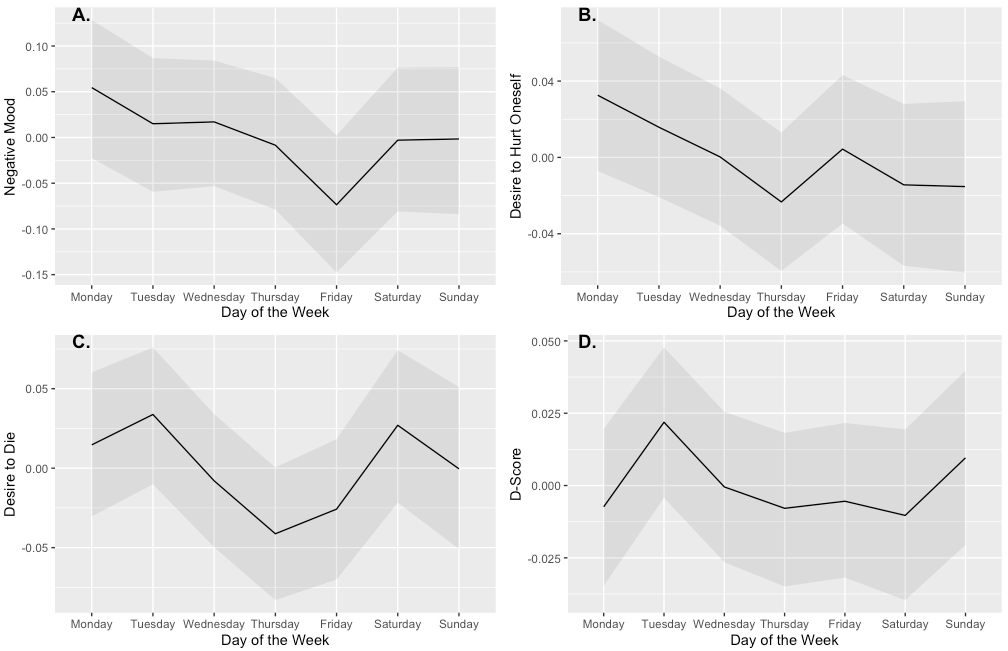


## Supplemental Section 5: Auto-ARIMA Models

**Table S4**

*Average number of observations used for monthly estimates in ARIMA models*

|  | Negative Mood | Desire to Die | Desire to Hurt Oneself | IAT D-Score |
| --- | --- | --- | --- | --- |
| Entire Sample | 132.22 | 132.24 | 132. 23 | 133.95 |
| Suicide Attempt | 41.33 | 41.45 | 41.44 | 41.63 |
| Self-Harm / Suicide Plan | 49.14 | 49.19 | 49.15 | 49.37 |
| No Self-Harm | 38.49 | 38.55 | 38.58 | 38.79 |

*Note.* The small differences in the average number of observations between the outcome variables are due to the removal of participants with missing observations. We always used the maximum number of observations for the respective analyses.

**Figure S12**

*Seasonal ARIMA Model Forecast for All Respondents for Negative Mood*

**
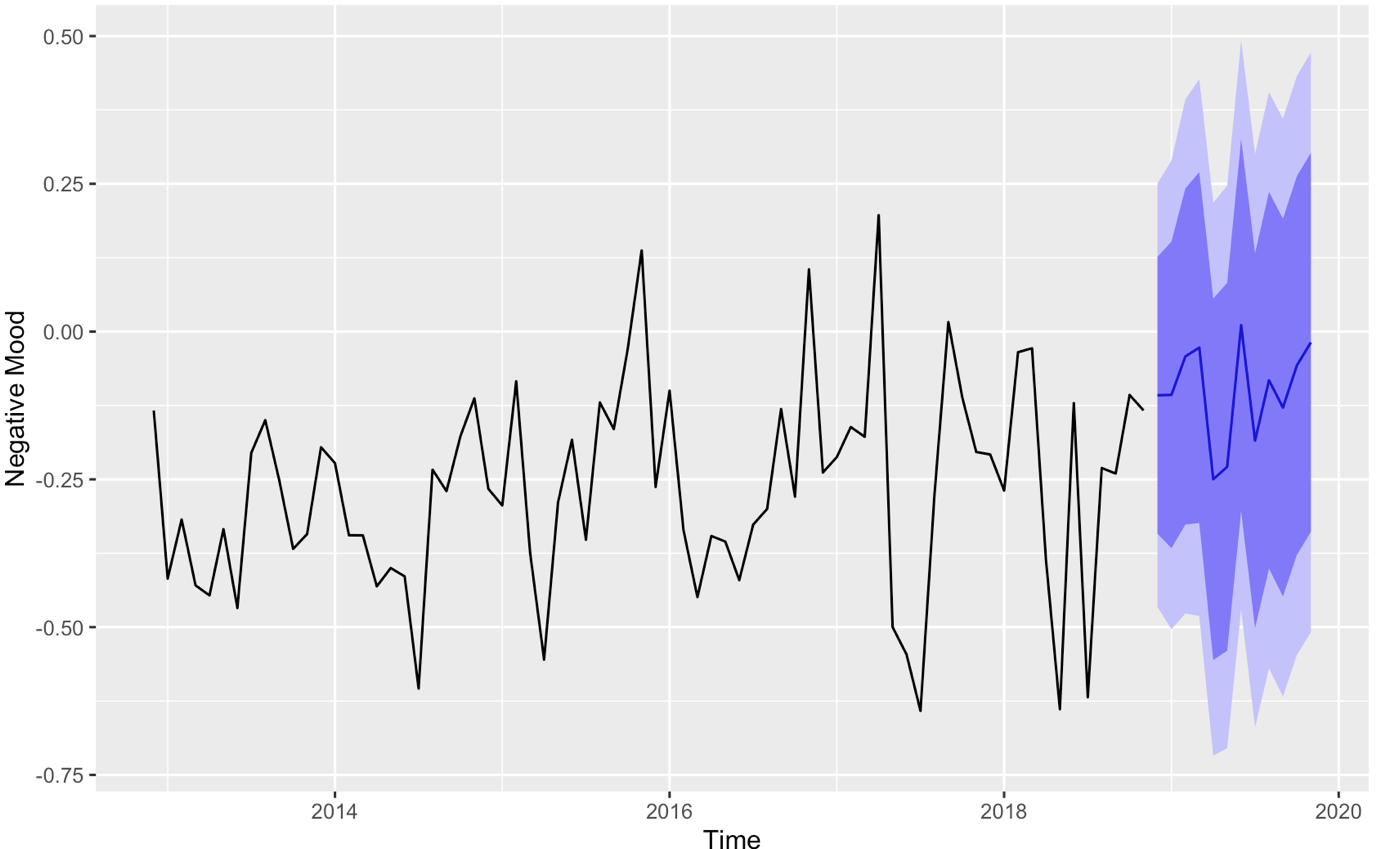
**

**Figure S13**

*Seasonal ARIMA Model Forecast for All Respondents for Desire to Die*

**
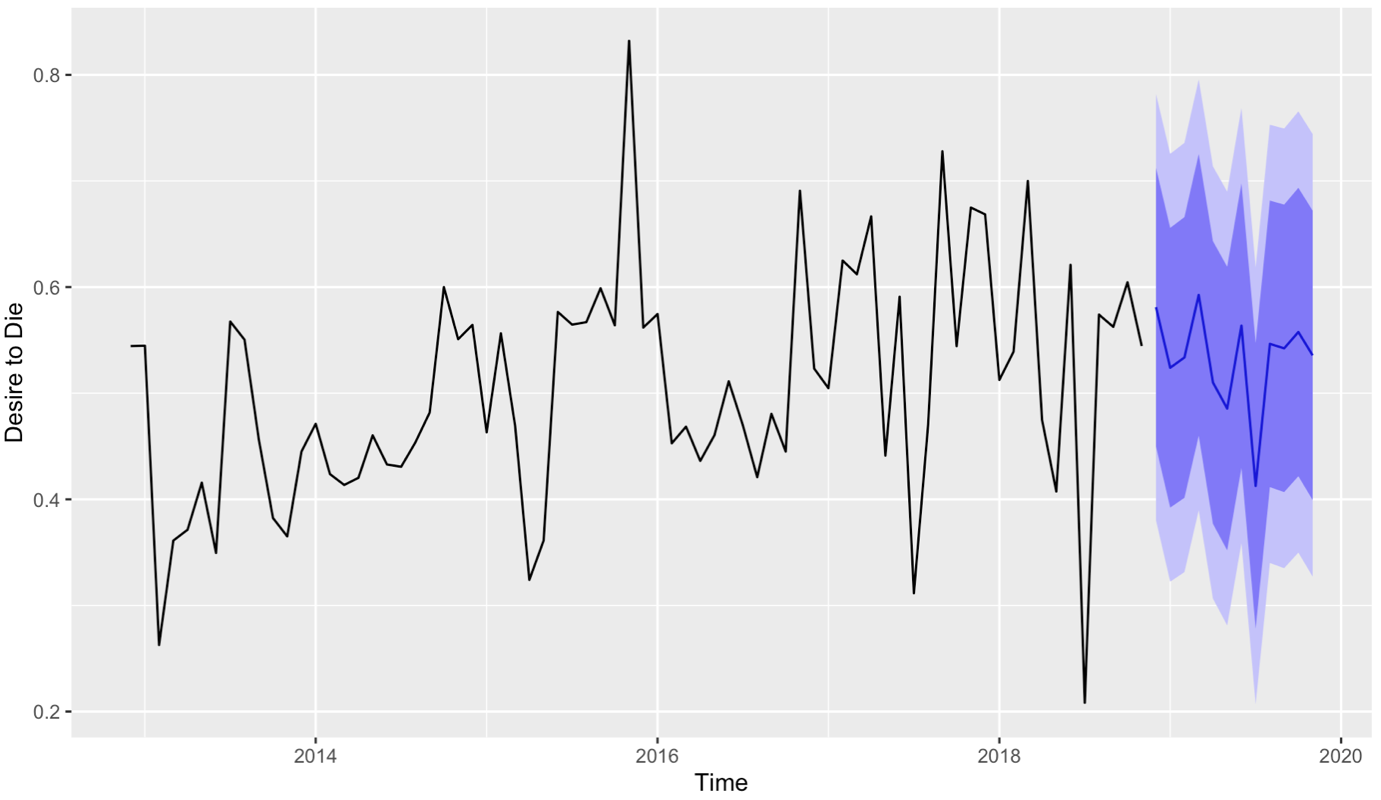
**

**Figure S14**

*Seasonal ARIMA Model Forecast for Respondents with a History of Suicide Attempts for Negative Mood*


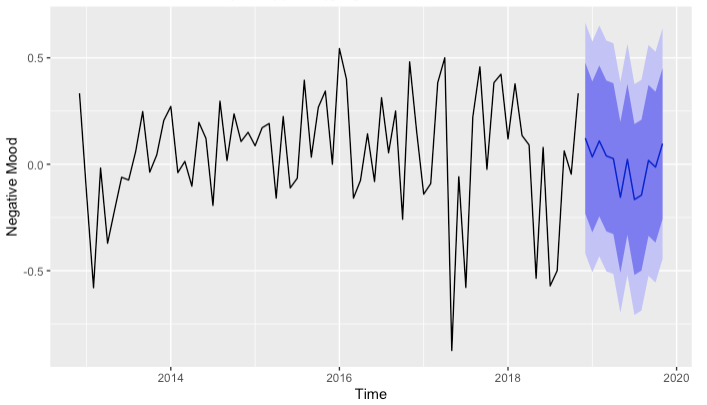


**Figure S15**

*Seasonal ARIMA Model Forecast for Respondents with a History of Suicide Attempts for Desire to Die*


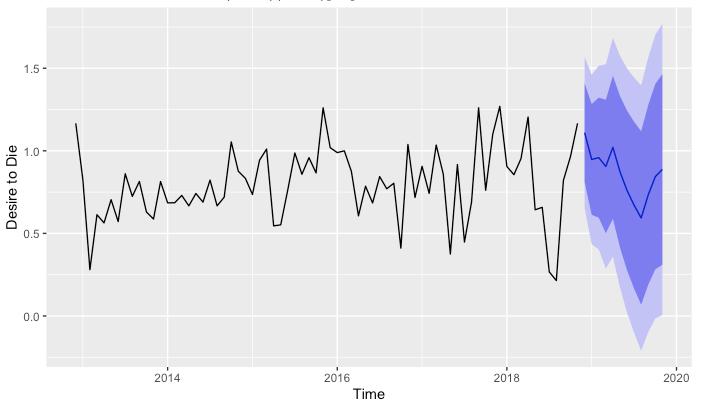


## Supplemental Section 6: Group Means and Comparisons

In addition to the ARIMA models, we examined the group means collapsed across all years and used two-sample t-tests as a convergent method. Consistent with both the ARIMA results and the decomposed Prophet forecast, the t-tests (see Table 1) showed significant differences between June and December for mood and desire to die in the group of respondents with a past suicide attempt. However, the less conservative t-tests also showed other significant effects (see Table 1).

**Figure S16**

*Averaged Means of all Outcome Scores for the Different Years in Combined Sample 1 and 2*


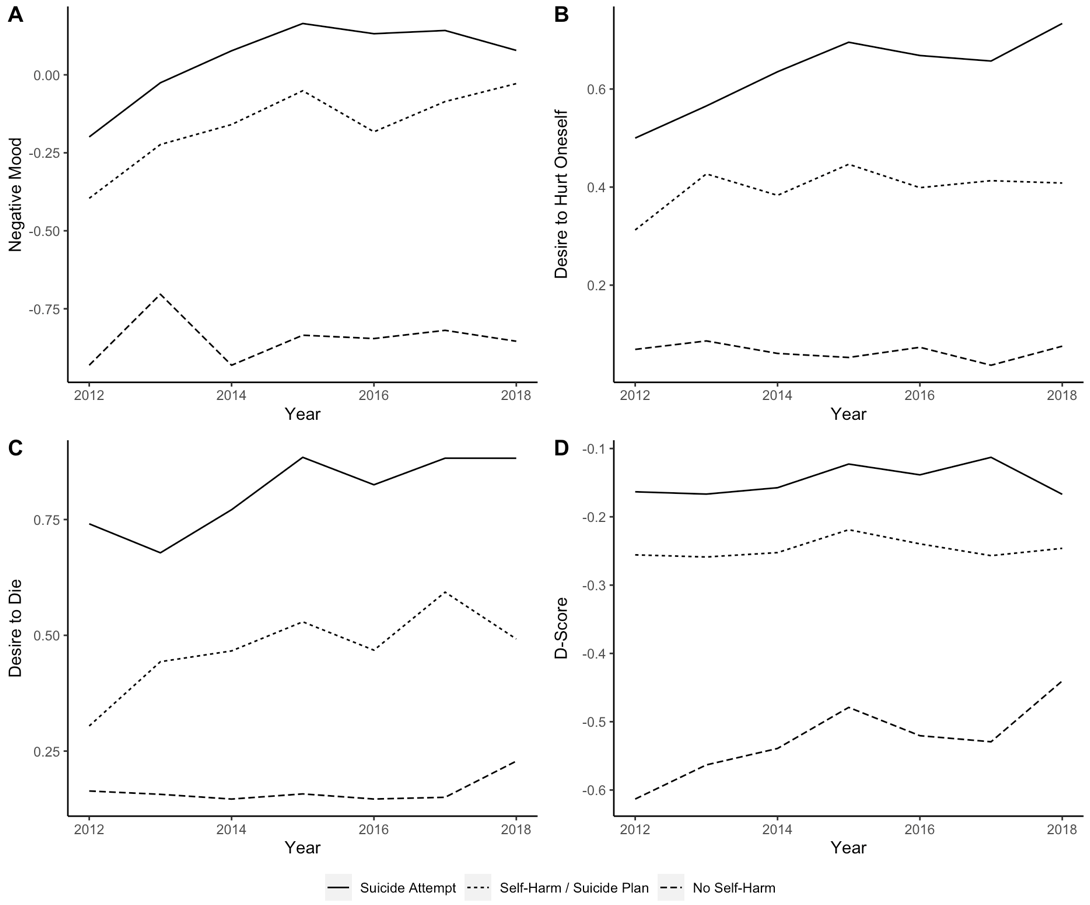


**Figure S17**

*Averaged Means of all Outcome Scores for the Months of the Year in the Sample with Accurate Time and Date Information*


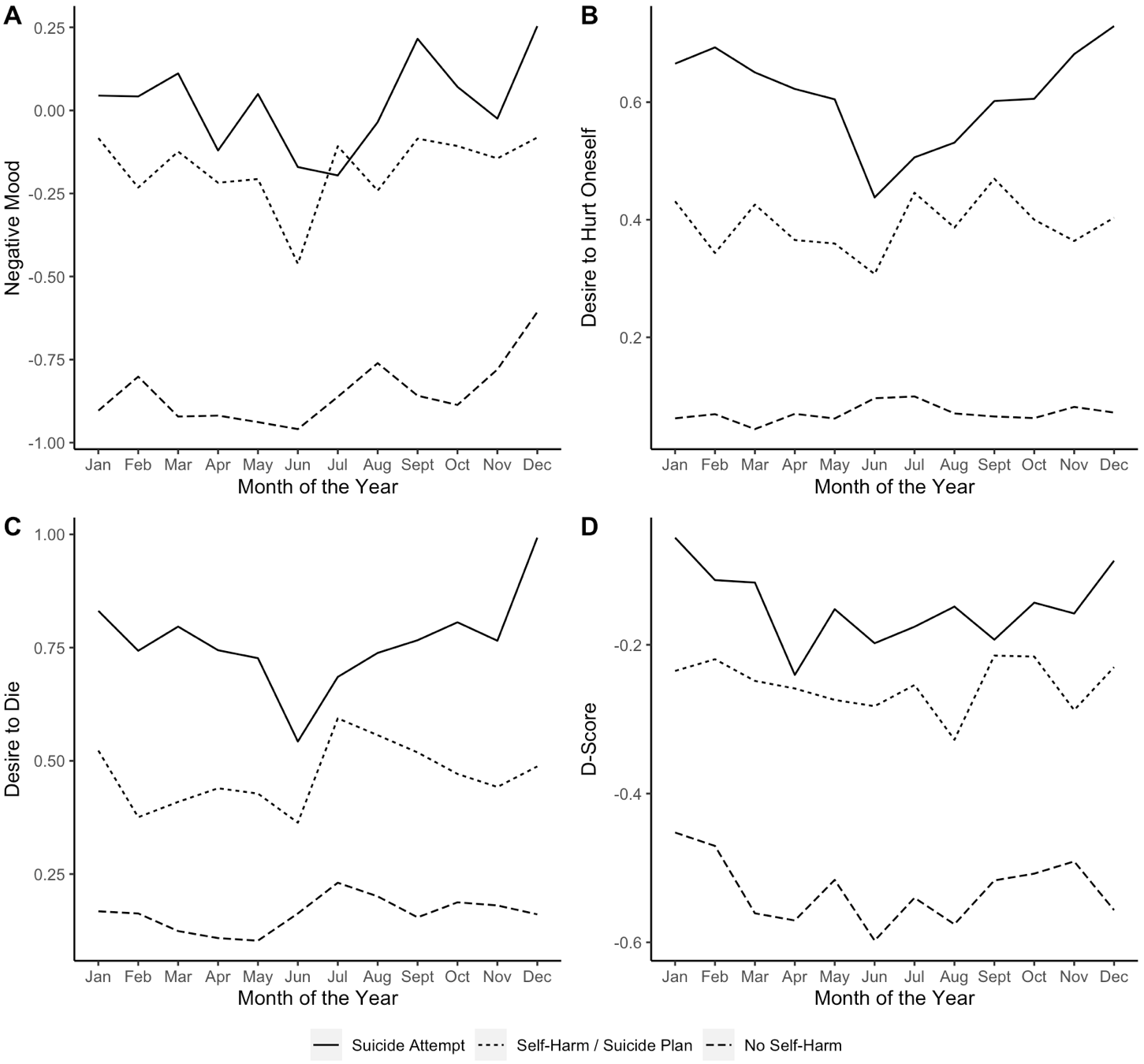


**Figure S18**

*Averaged Means of all Outcome Scores for the Day of the Week in the Sample with Accurate Time and Date Information*


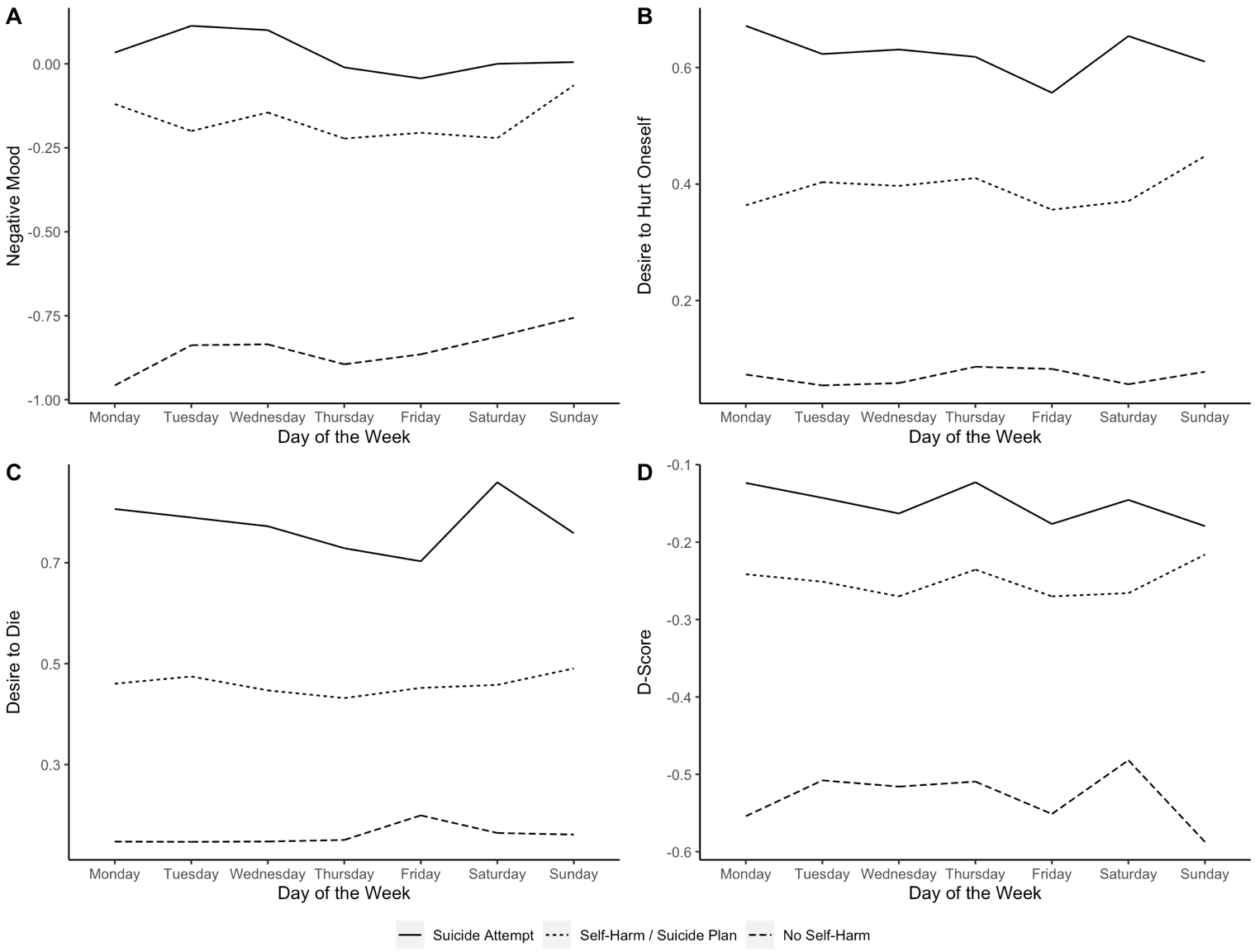


**Figure S19**

*Averaged Means of all Outcome Scores for the Time of the Day in the Sample with Accurate Time and Date Information*


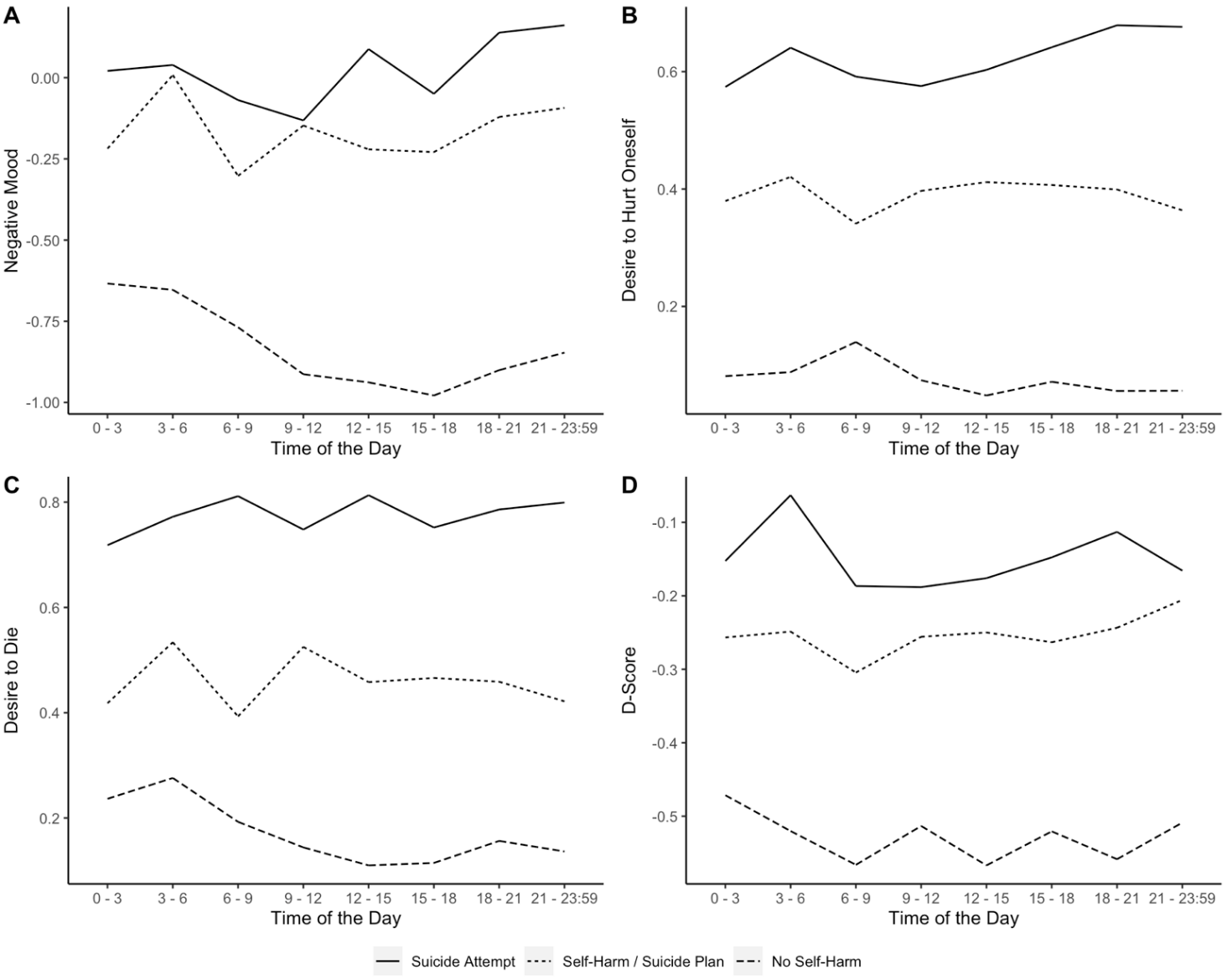


**Figure S20**

*Averaged Monthly Scores for Mood, Desire to Hurt Oneself, Desire to Die and the IAT D-Scores in all Groups*


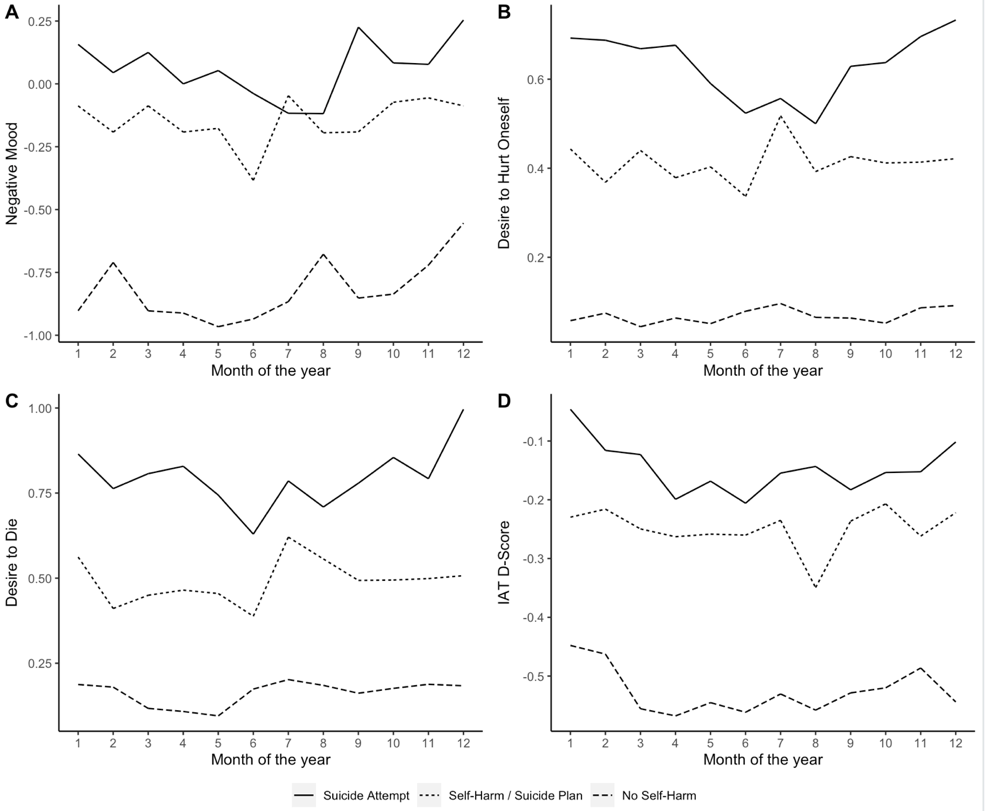


To examine whether the IAT type may impact our results, we have further separated the IAT types and examined the averaged IAT D-Score distribution as a function of month of the year. The results suggest that generally, the seasonal pattern (peak in December-February) is mostly consistent across IATs (see Figure S21).

**Figure S21**

*Averaged Monthly IAT D-Scores For Different IAT Types*


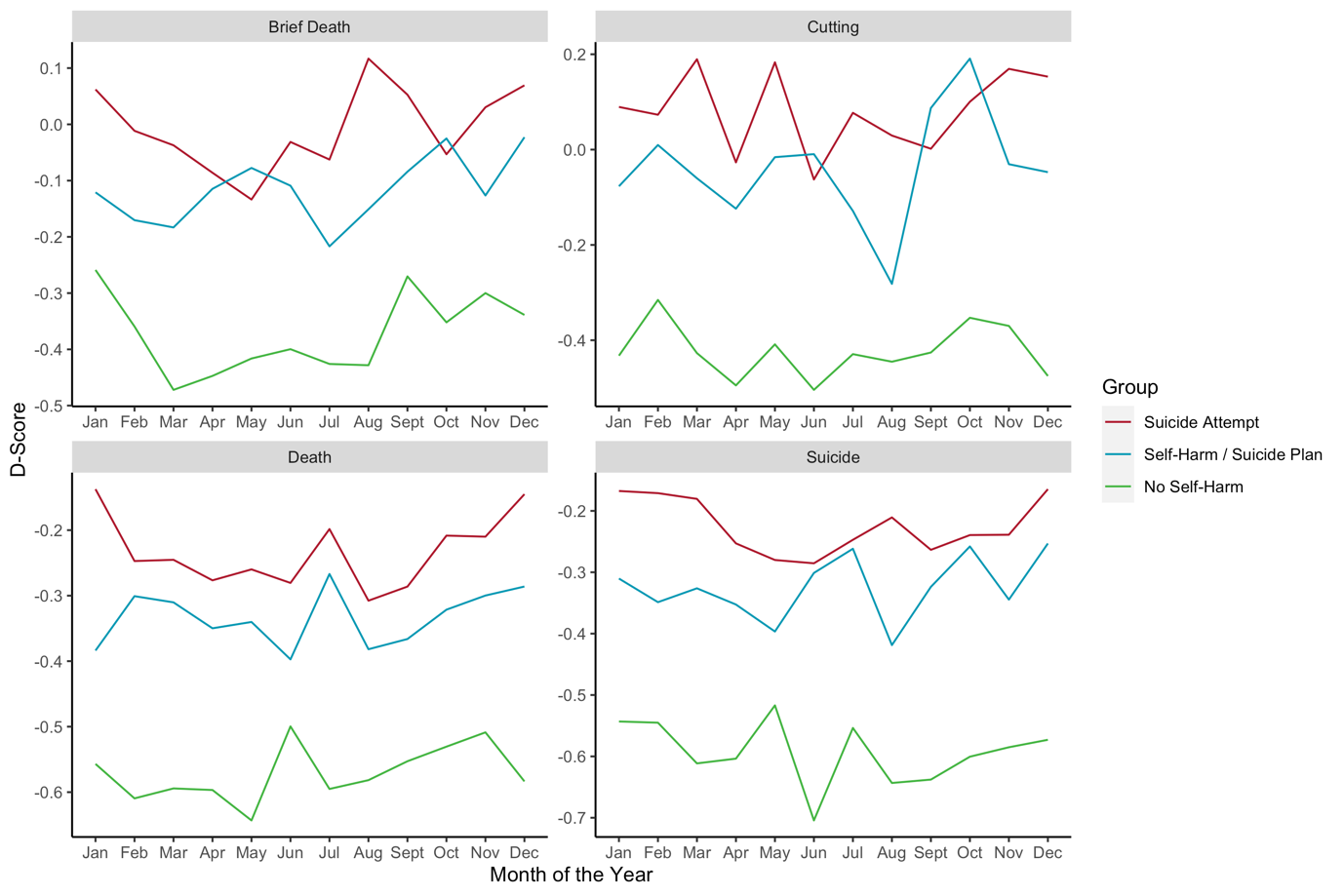


The groups used in the main manuscript are of similar size (suicide attempt: n = 3247; self-harm/suicidal ideation n = 3851, no self-harm n = 3026); thus, they allowed us to obtain robust monthly estimates and fair group comparisons. When we separated self-harm and suicidal ideation, then the separate groups are vastly different in size (only NSSI, no suicide plan: n = 2441; NSSI and suicide plan n = 1060; only suicide plan, no NSSI: n = 350). We examined monthly averages for these subgroups which are shown in Figure S22. We excluded respondents who only reported suicidal ideation due to too few observations (< 16) for certain months.

*Averaged Monthly Scores for Mood, Desire to Hurt Oneself, Desire to Die and the IAT D-Scores in Different Subgroups*


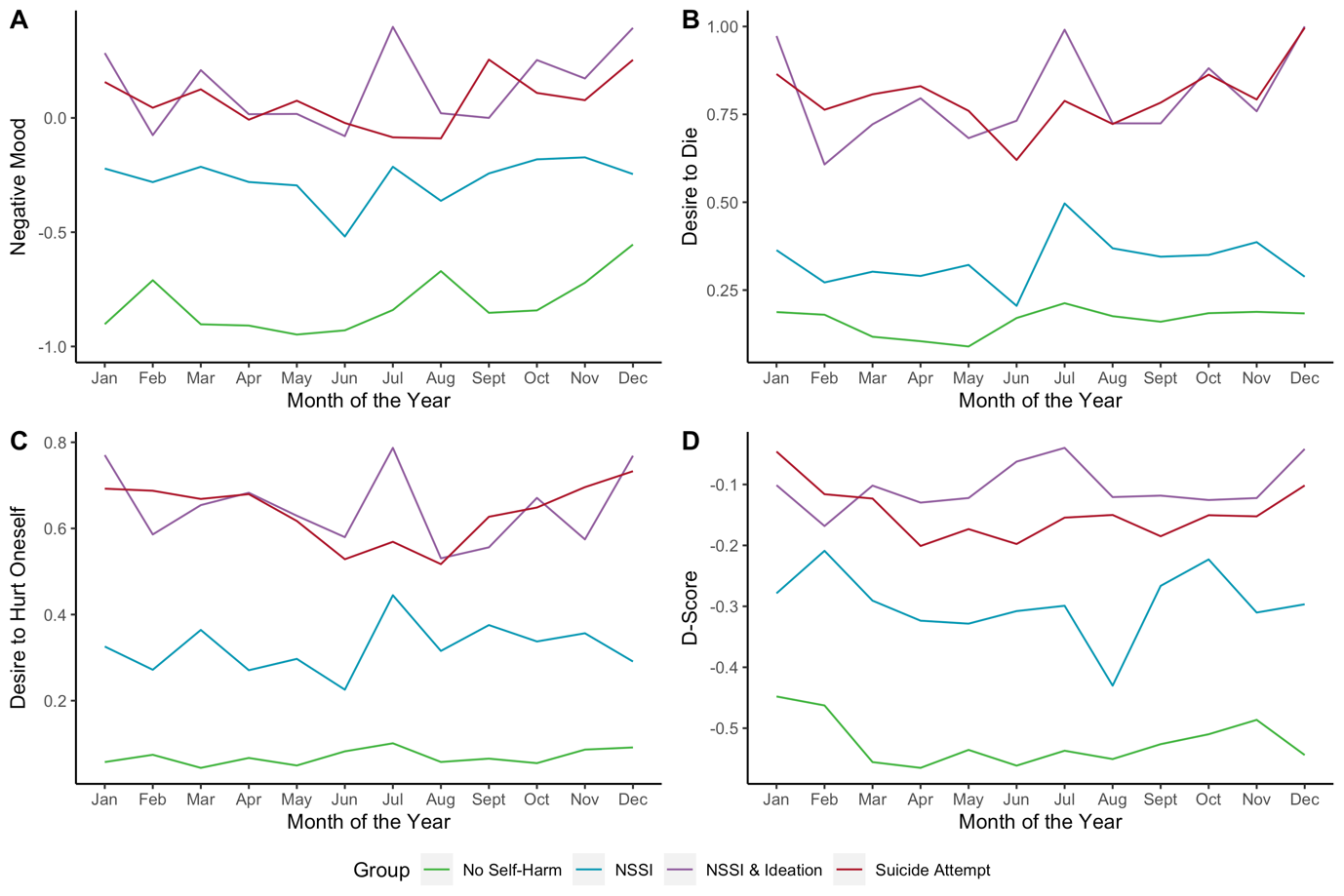


## Supplemental Section 7: Granger Models of Predictive Causality

**Table S5**

*Exploratory Granger Tests of Causality for Explicit Desire to Die and the Implicit IAT D-Score*

|  | Desire to Die precedes Implicit Association | | Implicit Association precedes Desire to Die | |
| --- | --- | --- | --- | --- |
| Desire to Die  Month  1 month  2 months  3 months  Weekday  1 day  2 days  Hour  1 hour  2 hours | *F-Value*  0.72  5.73  2.04  1.43  0.67  3.88  2.31 | *P-Value*  0.4  0.005**  0.12  0.23  0.51  0.04*  0.10 | *F-Value*  0.36  0.29  0.75  0.01  0.05  1.08  2.16 | *P-Value*  0.55  0.55  0.21  0.93  0.95  0.30  0.12 |

*Note.* The significance of the p-values is reported in asterisks (†*p*<.10, * ≤ 0.05; ** ≤ 0.01).

**Granger Causality Test for Desire to Die (Excluding the Cutting IAT)**

**Table S6**

*Exploratory Granger Tests of Causality for Explicit Desire to Die and the Implicit IAT D-Score Excluding the Cutting IAT*

|  | Desire to Die precedes Implicit Association | | Implicit Association precedes Desire to Die | |
| --- | --- | --- | --- | --- |
| Desire to hurt | *F-Value* | *p-Value* | *F-Value* | *p-Value* |
| Month |  |  |  |  |
| 1 month | *3.59* | *0.06*† | *2.25* | *0.14* |
| 2 months | *2.6* | *0.08*† | *3.86* | *0.03** |
| 3 months | *1.61* | *0.19* | *3.52* | *0.02* |
| Weekday |  |  |  |  |
| 1 day | *0.26* | *0.61* | *0.2* | *0.87* |
| 2 days | *0.14* | *0.87* | *2.37* | *0.09* |
| Hour |  |  |  |  |
| 1 hour | *3.37* | *0.07*† | *1.66* | *0.2* |
| 2 hours | *2.17* | *0.12* | *2.37* | *0.09*† |

*Note.* The significance of the p-values is reported in asterisks (†*p*<.10, * ≤ 0.05; ** ≤ 0.01). We removed the cutting IAT because the content is more different to Desire to Die than the content from the other three IATs. Additionally, we would not have enough power to run separate Granger models on each IAT type.

**Granger Causality Tests for Other Outcome Measures**

**Table S7**

*Exploratory Granger Tests of Causality for Explicit Desire to Hurt and the Implicit IAT D-Score*

|  | Desire to Hurt precedes Implicit Association | | Implicit Association precedes Desire to Hurt | |
| --- | --- | --- | --- | --- |
| Desire to hurt | *F-Value* | *p-Value* | *F-Value* | *p-Value* |
| Month |  |  |  |  |
| 1 month | *0.84* | *0.36* | *3.72* | *0.06*† |
| 2 months | *1.03* | *0.36* | *4.15* | *0.02** |
| 3 months | *2.00* | *0.12* | *1.84* | *0.15* |
| Weekday |  |  |  |  |
| 1 day | *3.98* | *0.047** | *0.39* | *0.53* |
| 2 days | *2.22* | *0.11* | *0.37* | *0.69* |
| Hour |  |  |  |  |
| 1 hour | *0.04* | *0.84* | *0.04* | *0.83* |
| 2 hours | *0.07* | *0.94* | *1.52* | *0.22* |

*Note.* The significance of the p-values is reported in asterisks (†*p*<.10, * ≤ 0.05; ** ≤ 0.01). All four IATs were included because we would not have enough power to detect an effect with only the cutting IAT in the analysis.

**Table S8**

*Exploratory Granger Tests of Causality for Negative Mood and the Implicit IAT D-Score*

|  | Negative Mood precedes Implicit Association | | Implicit Association precedes Negative Mood | |
| --- | --- | --- | --- | --- |
| Negative mood | *F-Value* | *p-Value* | *F-Value* | *p-Value* |
| 1 month | *4.07* | *0.047** | *4.27* | *0.04 ** |
| 2 months | *10.91* | *< 0.01*** | *7.44* | *0.02 ** |
| 3 months | *12.31* | *< 0.01*** | *12.86* | *< 0.01*** |
| Weekday |  |  |  |  |
| 1 day | *0.47* | *0.49* | *0.001* | *0.97* |
| 2 days | *0.998* | *0.37* | *0.4* | *0.67* |
| Hour |  |  |  |  |
| 1 hour | *2.81* | *0.09*† | *0.27* | *0.6* |
| 2 hours | *2.25* | *0.11* | *1.63* | *0.2* |

*Note.* The significance of the p-values is reported in asterisks (†*p*<.10, * ≤ 0.05; ** ≤ 0.01). When interpreting effects that are significant in both directions, a third variable may be driving these effects.

References

1. Greenwald AG, McGhee DE, Schwartz JL. Measuring individual differences in implicit cognition: the implicit association test. *J Pers Soc Psychol*. 1998;74(6):1464-1480. doi:10.1037//0022-3514.74.6.1464

2. O’Shea BA, Glenn JJ, Millner AJ, Teachman BA, Nock MK. Decomposing implicit associations about life and death improves our understanding of suicidal behavior. *Suicide Life Threat Behav*. 2020;50(5):1065-1074. doi:https://doi.org/10.1111/sltb.12652

3. Millner AJ, Coppersmith DDL, Teachman BA, Nock MK. The brief death implicit association test: scoring recommendations, reliability, validity, and comparisons with the death implicit association test. *Psychol Assess*. 2018;30(10):1356-1366. doi:10.1037/pas0000580

4. Greenwald AG, Nosek BA, Banaji MR. Understanding and using the implicit association test: i. an improved scoring algorithm. *J Pers Soc Psychol*. 2003;85(2):197-216. doi:10.1037/0022-3514.85.2.197

5. Glenn JJ, Werntz AJ, Slama SJK, Steinman SA, Teachman BA, Nock MK. Suicide and self-injury-related implicit cognition: a large-scale examination and replication. *J Abnorm Psychol*. 2017;126(2):199-211. doi:10.1037/abn0000230

6. Cha CB, Glenn JJ, Deming CA, et al. Examining potential iatrogenic effects of viewing suicide and self-injury stimuli. *Psychol Assess*. 2016;28(11):1510-1515. doi:10.1037/pas0000280

7. Nock MK, Holmberg EB, Photos VI, Michel BD. Self-injurious thoughts and behaviors interview: development, reliability, and validity in an adolescent sample. *Psychol Assess*. 2007;19(3):309-317. doi:10.1037/1040-3590.19.3.309

8. Benjamini Y, Hochberg Y. Controlling the false discovery rate: a practical and powerful approach to multiple testing. *J R Stat Soc Ser B Methodol*. 1995;57(1):289-300.

9. Charlesworth TES, Banaji MR. Patterns of implicit and explicit attitudes: i. long-term change and stability from 2007 to 2016. *Psychol Sci*. 2019;30(2):174-192. doi:10.1177/0956797618813087
